# Arabidopsis *HEAT SHOCK TRANSCRIPTION FACTORA1b* regulates multiple developmental genes under benign and stress conditions

**DOI:** 10.1093/jxb/ery142

**Published:** 2018-04-25

**Authors:** Waleed S Albihlal, Irabonosi Obomighie, Thomas Blein, Ramona Persad, Igor Chernukhin, Martin Crespi, Ulrike Bechtold, Philip M Mullineaux

**Affiliations:** 1 Department of Microbial & Cellular Sciences, Faculty of Health and Medical Sciences, University of Surrey, Guildford, Surrey, UK; 2 School of Biological Sciences, University of Essex, Wivenhoe Park, Colchester, Essex, UK; 3 Institute of Plant Sciences-Paris-Saclay, Gif-sur-Yvette Cedex, France; 4 Cancer Research UK Cambridge Institute, University of Cambridge, Cambridge, UK

**Keywords:** ChIP-seq, development, heat shock factors, long non-coding natural antisense RNAs, RNA-seq, stress, transcription factors

## Abstract

In *Arabidopsis thaliana*, *HEAT SHOCK TRANSCRIPTION FACTORA1b* (*HSFA1b*) controls resistance to environmental stress and is a determinant of reproductive fitness by influencing seed yield. To understand how *HSFA1b* achieves this, we surveyed its genome-wide targets (ChIP-seq) and its impact on the transcriptome (RNA-seq) under non-stress (NS), heat stress (HS) in the wild type, and in *HSFA1b*-overexpressing plants under NS. A total of 952 differentially expressed HSFA1b-targeted genes were identified, of which at least 85 are development associated and were bound predominantly under NS. A further 1780 genes were differentially expressed but not bound by HSFA1b, of which 281 were classified as having development-associated functions. These genes are indirectly regulated through a hierarchical network of 27 transcription factors (TFs). Furthermore, we identified 480 natural antisense non-coding RNA (*cis*NAT) genes bound by HSFA1b, defining a further mode of indirect regulation. Finally, HSFA1b-targeted genomic features not only harboured heat shock elements, but also MADS box, LEAFY, and G-Box promoter motifs. This revealed that HSFA1b is one of eight TFs that target a common group of stress defence and developmental genes. We propose that HSFA1b transduces environmental cues to many stress tolerance and developmental genes to allow plants to adjust their growth and development continually in a varying environment.

## Teaser

Plants must co-ordinate gene expression to balance growth and stress defence. Arabidopsis HSFA1b influences this by reconfiguring an extensive transcription regulatory network at the onset of heat stress.

## Introduction

The heat shock response (HSR) is a highly conserved cellular process, which is regulated at the transcriptional level by the heat shock transcription factors (HSFs; Ohama *et al.*, 2016). In their active state, HSFs form homo- and/or heterotrimers, accumulate in the nucleus, and activate transcription of stress-associated genes by binding to heat shock *cis*-elements (HSEs). HSEs are three inverted pentameric DNA repeats of 5'-nGAAn-3' on the promoters of stress genes (Scharf *et al.*, 2012; Jaeger *et al.*, 2014; Zheng *et al.*, 2016; Jacob *et al.*, 2017). Importantly, the functions of HSFs go beyond the HSR to regulating responses to diverse abiotic and biotic stresses and the modulation of cell growth and development (Evans *et al.*, 2007; Akerfelt *et al.*, 2010; Hashimoto-Torii *et al.*, 2014; Jacob *et al.*, 2017).

Plant HSFs differ from those in other eukaryotes in three ways. First, plant HSF families are larger (Baniwal *et al.*, 2004; Guo *et al.*, 2016). For instance, there are 21 HSFs in *Arabidopsis thaliana* (Arabidopsis) compared with only single HSFs in both yeast and fruitfly and four in mammals (Nover *et al.*, 2001; Akerfelt *et al.*, 2010; Scharf *et al.*, 2012; Guo *et al.*, 2016). Secondly, plant HSFs are divided into three structurally distinct classes: A, B, and C. Classes A and C function as transcription activators, whereas members of class B HSFs are transcriptional repressors (Scharf *et al.*, 2012; Guo *et al.*, 2016; Jacob *et al.*, 2017). Thirdly, in addition to post-translation regulation of HSFs, some plant *HSF* genes, such as *HSFA2*, *A3*, *B2a*, and *B2b*, are regulated transcriptionally in a stress-dependent manner by clade A1 *HSF* genes as well as other transcription factors (TFs; Schramm *et al.*, 2008; Scharf *et al.*, 2012; Bechtold *et al.*, 2013; Guo *et al.*, 2016; Jacob *et al.*, 2017). This has led to diversification of tissue and functional specificities (Liu *et al.*, 2011; Guan *et al.*, 2013; Hwang *et al.*, 2014; Perez-Salamo *et al.*, 2014; Jacob *et al.*, 2017). In Arabidopsis, the clade A1 HSF family, which is expressed in all tissues (Miller and Mittler, 2006, Swindell *et al.*, 2007; Bechtold *et al.*, 2013), consists of four genetically redundant members, *HSFA1a*, *HSFA1b*, *HSFA1d*, and *HSFA1e*. Only a quadruple null mutant *hsfA1a*/*hsfA1b*/*hsfA1d*/*hsfA1e* (hereafter called qKO) is unable to initiate HSR (Liu *et al.*, 2011; Yoshida *et al.*, 2011). Furthermore, qKO plants show diminished development and growth manifested throughout all growth stages. This is not evident in genotypes with fewer combinations of clade A1 HSF null mutants (Busch *et al.*, 2005; Liu *et al.*, 2011). The qKO mutant is defective in the development of the seed, consequently retarding germination, seedling establishment, and also growth through all vegetative stages. However, qKO mutant plants do complete their life cycle under non-stress (NS) conditions (Liu *et al.*, 2011).

Overexpression of *HSFA1b* in Arabidopsis and oilseed rape (*Brassica napus*) not only promotes enhanced resistance to abiotic and biotic stress but also affects development by causing a redistribution of biomass in favour of reproductive structures at the expense of vegetative shoot growth, resulting in increased seed yield (Prändl *et al.*, 1998; Bechtold *et al.*, 2013; Jung *et al.*, 2013). In contrast to the qKO mutant, there is no effect of *HSFA1b* overexpression on any aspect of seed or seedling viability (Bechtold *et al.*, 2013).

The identity of development-associated genes that are subject to regulation by clade A1 HSFs needs to be determined, as well as the full extent of those genes involved in defence against stress. Therefore, we set out to identify *HSFA1b*-regulated development-associated genes under NS and heat stress (HS) conditions and to determine how these genes may be regulated in comparison with stress-associated genes. By applying HS for a short period of 30 min, when HSFA1b is active (Busch *et al.*, 2005; Li *et al.*, 2010; Liu *et al.*, 2011), we anticipated that we would detect not only the early events in the induction of stress-defensive genes but also changes in expression of genes implicated in growth and development. This was done by surveying genome-wide binding of HSFA1b to its target genes and combining this with transcriptomics using both wild-type (WT) plants under NS and HS conditions, and those overexpressing *HSFA1b* under NS conditions.

## Materials and methods

### Arabidopsis genotypes and transgenes

The 35S:HSFA1b plants (35S:HSFA1b-RFP-B/Col-0), the *hsfA1a/hsfA1b* (Ws-0) mutant, and the qKO mutant (Col-0/Ws-0) have been described previously (Busch *et al.*, 2005; Liu *et al.*, 2011; Bechtold *et al.*, 2013). To construct the HSFA1b^PRO^:*HSFA1b-eYFP* (enhanced yellow fluorescent protein) gene fusion (hereafter called NP:HSFA1b), *HSFA1b* was PCR-amplified, using Phusion DNA polymerase (Thermo-Fisher, Paisley, UK), from Arabidopsis Col-0 genomic DNA, generating an amplicon containing its promoter and full genomic sequence minus the stop codon using primers 5'-CACCTCGAATAATTGTCAAGCTCAC-3' and 5'-TTTCCTCTGTGCTTCTGAG-3'. The amplicon was inserted into the pENTR plasmid using the D-TOPO cloning kit (Thermo-Fisher). The Gateway LR reaction (Thermo-Fisher) was used to create the *HSFA1b-eYFP* fusion in the binary Ti vector pGWB40 (Nakagawa *et al.*, 2007) creating pGWB40-HSFA1b^PRO^:*HSFA1b*-eYFP, which was transferred to *Agrobacterium tumefaciens* strain GV3101 and used to transform Arabidopsis Col-0 (Bechtold *et al.*, 2013). Transformants were selected on Murashige and Skoog (MS) medium containing 25 µg ml^–1^ hygromycin B and 50 µg ml^–1^ kanamycin. To select expressing lines, immunoblotting (Prändl *et al.*, 1998) was carried out using anti-green fluorescent protein (GFP) antibody (ab209; Abcam, Cambridge, UK).

### Growth phenotypes and HS conditions

All plants were grown in an 8 h day:16 h night under controlled environment conditions (Bechtold *et al.*, 2013). Five-week-old plants were subjected to HS at 37 °C (from 23 °C) for 30 min at 86% relative humidity to maintain the vapour pressure deficit at 1 kPa and therefore avoid a coincident dehydration stress (Fryer *et al.*, 2003; Galvez-Valdivieso *et al.*, 2009).

Rosette expansion of soil-grown 2-week-old seedlings over 11 d was carried out to monitor the effect of HS (2 h at 37 °C) or overexpression of HSFA1b under non-stress conditions. Daily measurements of rosette area were made using a chlorophyll fluorescence imager (Fluorimager, Technologica Ltd, Colchester, UK) as described by Baker (2008).

### ChIP-PCR, ChIP-seq, and data analysis

Fully expanded leaf samples from 5-week-old NS and HS NP:HSFA1b and Col-0 plants were used for ChIP experiments. A detailed step-wise protocol can be found as a Supplementary Methodology at *JXB* online. PCR analysis to detect *HSFB2a* in ChIP DNA samples was carried out as previously described (Bechtold *et al.*, 2013). Library preparation and massively parallel DNA sequencing of ChIP DNA samples (ChIP-seq) were carried out at The Genome Analysis Centre (now the Earlham Institute; http://www.earlham.ac.uk/) using Illumina TruSeq ChIP Library Prep Kit (Illumina, San Diego, CA, USA) according to the manufacturer’s instructions. Libraries were sequenced on an Illumina HiSeq2000 platform using 100 bp paired ends and a sequencing depth of ≥10 million reads per library. The data from two independent plants in one HS experiment are combined.

Quality control of raw fastq files was carried out using in-house programs (available upon request). Using GSNAP (Wu and Nacu, 2010), ChIP-seq reads were mapped to the Arabidopsis genome (TAIR10) allowing one mismatch and output files in SAM format. Removal of unmapped reads and duplicates followed by conversion of SAM format to the binary form (BAM), sorting and indexing of BAM files was done using SAMtools (Li *et al.*, 2009). Library normalization followed by conversion to signal tracks in BedGraph format was performed using BEDtools (Quinlan and Hall, 2010). Normalized BedGraph files were visualized using the Integrated Genome Browser (IGB; Nicol *et al.*, 2009). Peaks were called using MACS2 (Zhang *et al.*, 2008) with the options –g 1.2e8 –f BAMPE –q 0.05 –B –trackline –FE 2. Peaks within pericentromeric regions and broad peaks on gene bodies of highly transcribed genes were considered as false positives regardless of their *q* and FE values (Nix *et al.*, 2008; Chen *et al.*, 2012). *K*-means clustering of ChIP-seq signals on the regions occupied by HSFA1b and generation of density heat maps were carried out using seqMINER (Ye *et al.*, 2011). Annotation of the closest genomic features to the regions bound by HSFA1b was carried out with ChIPpeakAnno (Zhu *et al.*, 2010) using the batch annotation function and a dedicated function for peaks on bidirectional promoters. Overlap between annotated target genomic features was determined and Venn diagrams were generated using Jvenn (Bardou *et al.*, 2014). Gene Ontology (GO) analysis on the target genomic features was carried out using the Singular Enrichment Analysis (SEA) tool in the AgriGO database (Du *et al.*, 2010). Sequences of the regions bound by HSFA1b were used for *de novo* motif discovery using MEME (Bailey *et al.*, 2009) with a cut-off *P*<0.0001 and using default options.

The published ChIP-seq data analysed in this study were downloaded from the NCBI Gene Expression Omnibus (GEO) database and subjected to the same analysis using MACS2 and criteria as described above. The GEO accession codes for each TF are as follows: HBI1, GSE53078; LFY, GSE24568; PIF5/PIF4, GSE68193; PRR5, GSE36361; PRR7, GSE49282; and SEP3, GSE46987.

### RNA-seq data analysis

RNA was extracted from NS WT, HS WT, and NS 35S:HSFA1b plants (three biological replicates of each) as described previously (Bechtold *et al.*, 2013) and analysed using massively parallel sequencing (RNA-seq) as follows: synthesis of cDNA, library preparation, and sequencing were carried out at Earlham Institute on the Illumina HiSeq2000 platform using 50 bp paired-end sequencing with minimum read depth ≥25 million reads per library.

Quality control of raw fastq files was carried out as for the ChIP-seq data with the following modifications: filtered RNA-seq reads were mapped against the Arabidopsis transcriptome using GSNAP with the known splices option for RNA-seq (five mismatches allowed). Transcript assembly and differential expression analysis were carried out using Cufflinks and Cuffdiff (Trapnell *et al.* 2012) followed by the geometric library normalization method with threshold *q*≤0.05.

Assembly of a hierarchical TF network was done by downloading all connections for selected TFs from the Cistrome Atlas (O’Malley *et al.*, 2016) or BZIP28 ChIP-seq data (Zhang *et al.*, 2017) and assembling all pairwise interactions in Excel manually in the form Gene A (PD) Gene B, using an exemplar template available from a previously published analysis (Bechtold *et al.*, 2016). The resulting file containing the connections was uploaded into Cytoscape 3.3.1 (Shannon *et al.*, 2003; www.cytoscape.org) and visualized initially using default settings.

### Identification of lincRNAs and *cis*NAT RNAs

The sequenced reads were aligned using TopHat 2 (Kim *et al.*, 2013) on the TAIR10 DNA sequence. The GFFProf script included with RNAprof (Tran *et al.*, 2016) was used to predict all new transcriptional units compared with Araport11 gene annotations (Cheng *et al.*, 2017). Only the transcriptional units >200 nucleotides were kept. The coding potential was estimated using existing annotation (repTAS; Liu *et al.*, 2012) and CANTATAdb (Szcześniak *et al.*, 2016) and, if absent, it was predicted using COME (Hu *et al.*, 2017). Using R (http://www.R-project.org/), all annotated target genomic features of HSFA1b were intersected with transcribed genomic features to generate normalized FPKM (fragments per kilobase of transcript per million mapped reads) values. FPKM values=0 in all conditions were discarded. The differential expression of HSFA1b target genes was determined based on *q*-value and fold change. Genes were designated as up-regulated and down-regulated based on expression values of HSFA1b target genes in the WT under HS and 35S:HSFA1b under NS relative to NS WT.

### qRT-PCR

The method and primers for *APX2*, *MBF1c*, *HSFA2*, and *HSFB2b* have been described previously (Bechtold *et al.*, 2013). All other primers used in this study are given in Supplementary Table S2.

### Accession number

The ChIP-seq and RNA-seq data have been deposited in the NCBI GEO database under code GSE85655.

## Results

### HSFA1b preferentially binds to downstream and intragenic regions of its target genes under NS conditions

Genome-wide HSFA1b target genes were identified by ChIP-seq from NP:HSFA1b plants expressing a C-terminal fusion of HSFA1b to eYFP under the control of its native promoter (see the Materials and methods). C-terminal fusions to clade A1 HSFs, including HSFA1b, do not affect their function (Prändl *et al.*, 1998; Liu *et al.*, 2008; Jung *et al.*, 2013; Bechtold *et al.*,2013). The transgenic line chosen (NP:HSFA1b_6) had the least immunodetectable protein of the seven lines surveyed (Supplementary Fig. S1A) and was validated as suitable for ChIP-seq by carrying out ChIP-PCR to show binding of HSFA1b–eYFP to the promoter of *HSFB2b* (Supplementary Fig. S1B, C; see the Materials and methods). ChIP-seq was performed on 5-week-old plants grown under NS conditions and subjected to HS treatment (37 °C for 30 min). The control for these experiments was Col-0 (WT) plants treated in the same way. *HSFA1b*, along with *HSFA1a*, regulates the initial phase (<1 h) of the HSR; thereafter, stress-inducible *HSF* genes take over (Busch *et al.*, 2005; Li *et al.*, 2010; Liu *et al.*, 2011). This was confirmed by a ≥30 min delay of HS-inducible gene expression in *hsfA1a/hsfA1b* compared with WT plants (Supplementary Fig. S2).

The minimum exposure to 37 °C that affected growth was 2 h, at which a slight but significant (*P*≤0.05; Student’s *t*-test) reduction in the rate of rosette expansion was measured 4 d and 5 d post-stress in Col-0 (Supplementary Fig. S3).

**Fig. 1.**
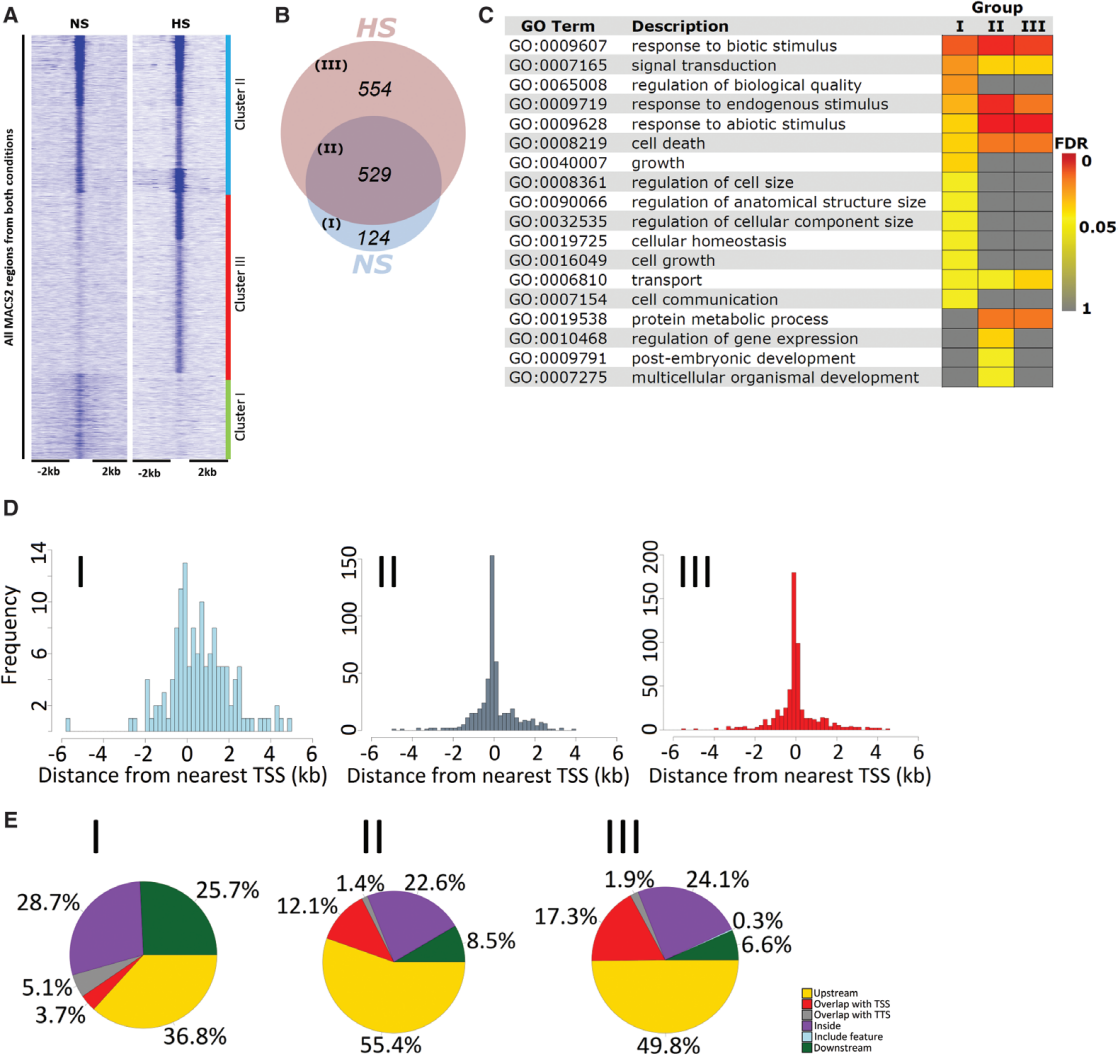
Alteration of HSFA1b binding in response to HS. (A) Heat map with *k*-means clustering showing the enrichment of ChIP-seq signals from NS and HS samples at a 4 kb window around HSFA1b-bound regions in the genome. (B) Venn diagram showing the number of HSFA1b target genomic features in Groups I–III. (C) GO Slim analysis heat map comparing genomic features of enriched Biological Process terms in Group I–III with Benjamini–Hochberg FDR <0.05. (D) Three histograms showing the frequency of HSFA1b binding relative to the distance from the TSS of target genomic features in Groups I–III. (E) Pie charts showing the distribution of HSFA1b binding on target genomic features in Groups I–III.

**Fig. 2.**
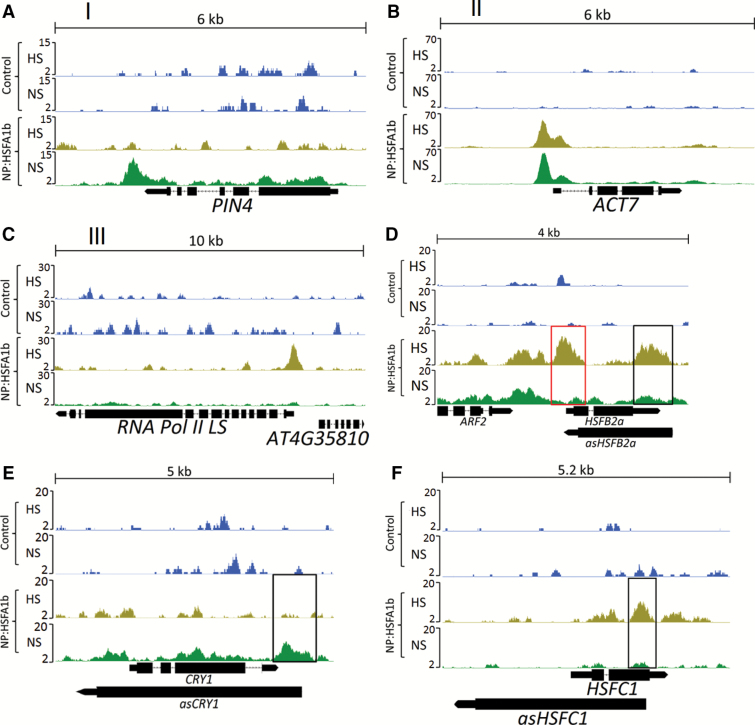
Genome browser view of normalized ChIP-seq tags in NP:HSFA1b NS and NP:HSFA1b HS along with controls showing examples of HSFA1b binding to genes in (A) Group I, (B) Group II, and (C) Group III. (D) Examples of HSFA1b targeting the promoter of *HSFB2a* (red rectangle) as well as a region known to code for an antisense RNA that targets *HSFB2a* (*asHSFB2a*; black rectangle). (E and F) HSFA1b targeting a region that codes for *asCRY1* and *asHSFC1*, respectively.

Peak calling from ChIP-seq data (see the Materials and methods) identified 709 and 1083 HSFA1b-binding sites [*q*≤0.05; fold enrichment (FE) ≥2] under NS and HS, respectively (Supplementary Data S1), comprising 1207 HSFA1b target genes. *K*-means clustering of the binding regions identified three groups (Fig. 1A; Supplementary Data S1): unique to NS (Group I), common to NS and HS (Group II), and unique to HS (Group III; Fig. 1B). Examples of HSFA1b binding near genomic features in Groups I–III are shown in Fig. 2A–C. The target genes in each group (Supplementary Data S1) were intersected with loci mapped to genome-wide DNase I-hypersensitive sites in NS and HS seedlings (Supplementary Fig. S4; Sullivan *et al.*, 2014). Open chromatin in NS conditions showed enrichment for Group I genes (*P*=4.31E-08) but less so for Group II and III genes (*P*=0.03 for both comparisons), while the opposite was observed for HS seedlings (Group I; *P*=0.91; Supplementary Fig. S4).

To obtain an overview of the biological functions of proteins encoded by HSFA1b-bound genes in each group, we carried out a GO analysis (see the Materials and methods). In all groups, there was a significant enrichment for Biological Process (BP) GO terms that reflected the role of HSFA1b in plant stress responses (Fig. 1C; Supplementary Data S2). However, there were many enriched BP terms associated with growth and development from Groups I and II but not Group III (Fig. 1C; Supplementary Data S2).

Forty percent of HSFA1b binding events mapped to within ~250 bp of the transcription start site (TSS) of Group II and III genes (Fig. 1D). In contrast, binding of Group I genes by HSFA1b was spread across greater distances (<1% of the binding sites mapped within 250 bp of the TSS; Fig. 1D). A breakdown of binding regions in relation to the main features of genes showed that HSFA1b preferentially targeted inside and downstream of genes in Group I (54%) in contrast to ~30% for Group II and III genes (Figs 1E; 2B, C; Supplementary Data S1).

### Detection of long non-coding RNAs

**Fig. 3.**
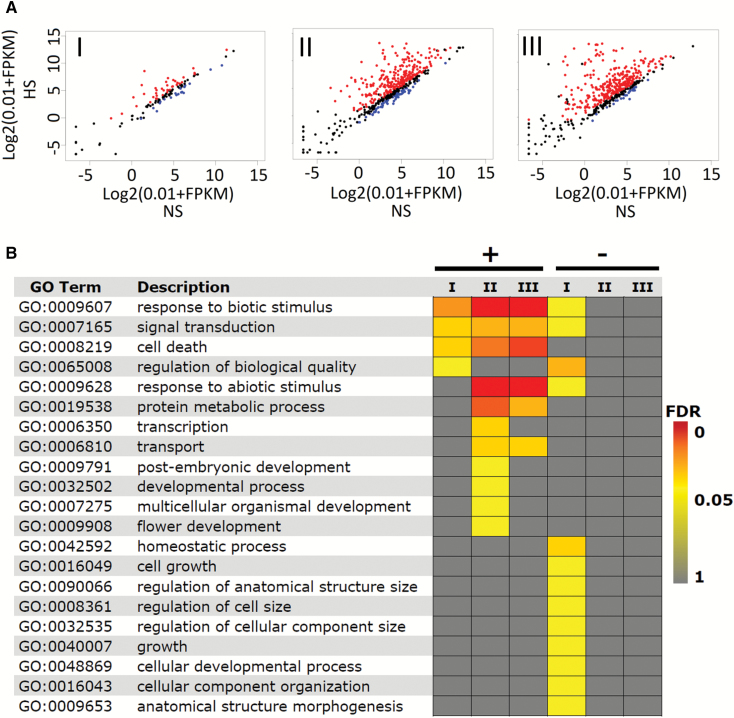
Most HSFA1b target genes are responsive to HS. (A) Scatter plots of transcript abundance of HSFA1b target genes in response to HS. Red and blue dots represent up- and down-regulated genes, respectively (*q*≤0.05), and black dots show genes with expression *q*>0.05. The numbers shown in each panel are the number of DEGs in each group. (B) GO Slim analysis heat map comparing the enriched BP terms of up- (+) and down- (–) regulated HS-responsive genes in Groups I–III (Benjamini–Hochberg FDR <0.05).

In WT plants under NS and HS, 7137 differentially expressed genes (DEGs; *q*-value ≤0.05) were identified in response to HS (Supplementary Data S3). Of these, 721 were HSFA1b-bound genes (Fig. 3A; Supplementary Data S4). Enriched GO terms revealed that down-regulated HS-responsive genes were enriched for a number of growth functions (Fig. 3B; Supplementary Data S5).

The prevalence of HSFA1b binding internal to and downstream of protein-coding genes suggested that it binds to genomic regions in or near *cis* natural antisense long non-coding (*cis*NAT) RNA genes (Ariel *et al.*, 2015). Using the RNA-seq data on WT (NS and HS) plants, we predicted all transcripts in addition to those present in the Araport11 database (Cheng *et al.*, 2017). The transcripts were then classified as coding transcripts, *cis*NAT or long intergenic non-coding (linc) RNAs (see the Materials and methods). All binding sites of Groups I–III classified as located internal or distal to the putative sense target gene (*cis* natural antisense long non-coding; Supplementary Data S1) were intersected with the complete list of NAT gene co-ordinates. Table 1 summarizes this analysis and includes data on the number of NAT genes that also have HSFA1b bound to the corresponding sense gene under NS and/or HS conditions (see also Fig. 2D–F).

**Table 1.** Summary of HSFA1b binding to *cis*NAT genes and lincRNA genes under NS and HS conditions (*q*-value <0.05)

	Group I (NS only)	Group II (HS and NS)	Group III (HS only)
**All *cis*NAT genes bound by HSFA1b (*n***)	76	364	377
**Proportion of *cis*NAT genes whose target sense gene is not bound by HSFA1b under the same conditions**	57%	58%	99%
**All lincRNA genes bound by HSFA1b (*n***)	11	39	29

### The transcriptome of *HSFA1b*-overexpressing plants shows an intermediate state between NS and HS wild-type plants

**Fig. 4.**
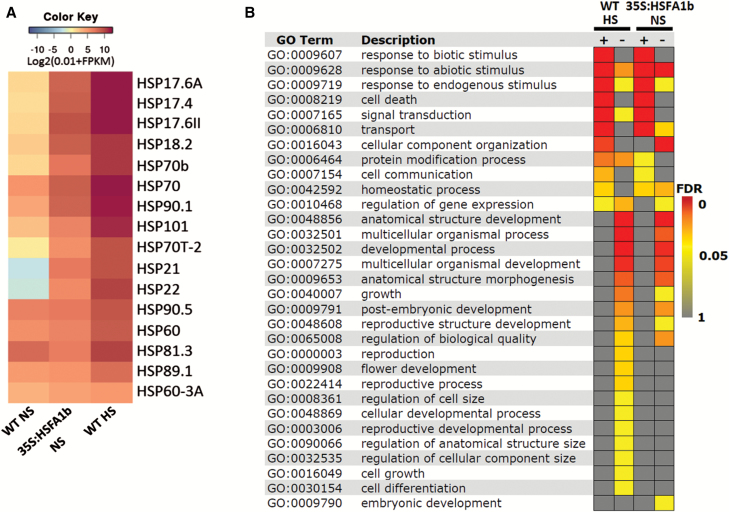
35S:HSFA1b plants under NS partially mimic HS WT plants. (A) Heat map comparing normalized FPKM values for HSP genes in NS and HS WT and 35S:HSFA1b NS plants. (B) GO Slim analysis comparing the enriched Biological Process terms (Benjamini–Hochberg FDR <0.05) of up- (+) or down (–)-regulated DEGs in HS WT and NS 35S:HSFA1b plants compared with NS WT plants.

We performed RNA-seq on 35S:HSFA1b plants overexpressing *HSFA1b-RFP* (see the Materials and methods) under NS conditions. A total of 3306 protein-coding genes showed differential expression in these plants compared with NS WT plants (*q*≤0.05; Supplementary Data S3), of which 72% were differentially expressed in HS WT plants (Supplementary Data S3). A Pearson correlation test on all the transcriptome data (*q*≤0.05) showed significant positive correlation between NS 35S:HSFA1b and both NS (*r*=0.92) and HS WT plants (*r*=0.88). Moreover, the expression levels of heat shock protein (HSP) genes in 35S:HSFA1b NS plants was intermediate to WT NS and HS plants (Fig. 4A). This suggested that the 35S:HSFA1b plants under NS conditions were poised in a state between growth and stress defence. Consistent with these observations, DEGs in 35S:HSFA1b NS plants showed enrichment for both stress-associated and developmental GO terms, paralleling the enriched BP terms in WT HS plants (Figs 3B, 4B; Supplementary Data S5). In keeping with our previous observations (Bechtold *et al.*, 2013), the rate of rosette expansion over 14–25 d post-germination was markedly reduced in 35S:HSFA1b compared with Col-0 (Supplementary Fig. S5A). At 5 weeks old, rosettes were typically visibly smaller (Supplementary Fig. S5B, C) and flowering time was shorter by 1 d (Supplementary Fig. S5D).

### *HSFA1b* extends its influence by exerting indirect control over gene expression

**Fig. 5.**
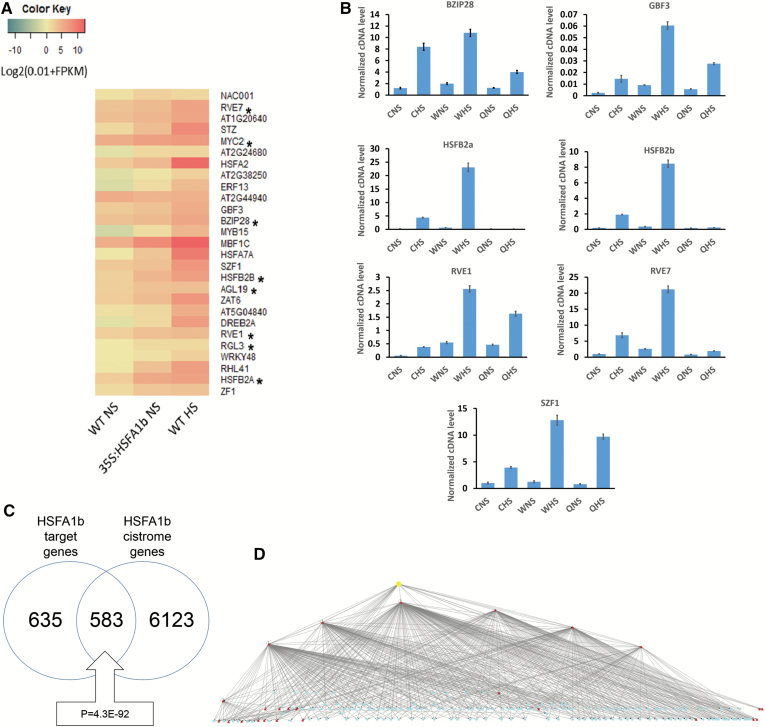
HSFA1b can indirectly control expression through its regulation of TF gene expression. (A) Heat map comparing normalized FPKM values for 28 TF genes bound by HSFA1b and differentially expressed in 35S:HSFA1b and WT HS plants. Asterisks (*) indicate development-associated TF genes. (B) Confirmation of the regulation of selected TF genes by clade A1 HSF genes. Quantitative real-time RT–PCR was conducted on RNA from qKO rosettes under NS and HS in comparison with its parental genotypes Col-0 (C) and Ws-0 (W). The suffixes ‘a’ and ‘b’ are where the qKO mutant shows a significant difference (*P*<0.05) under the same conditions (NS or HS) from Col-0 and Ws-0, respectively. (C) Venn diagram showing the overlap between HSFA1b target genes scored from the ChIP-seq data (Supplementary Data S1) and the target genes bound by HSFA1b (HSF3) from the Arabidopsis Cistrome Atlas (http://neomorph.salk.edu/dev/pages/shhuang/dap_web/pages/index.php). The boxed callout number is the *P*-value for the significance of the overlap between the two data sets (hypergeometric distribution test). (D) An overview of a Cytoscape-generated HSFA1b hierarchical TF gene network using the data outputs from the Cistrome Atlas with the ChIP-seq and RNA-seq data from this study. The yellow node is HSFA1b, red nodes are TF genes bound by HSFA1b, and blue nodes are differentially expressed TF genes that respond to HS and HSFA1b overexpression, are not bound by HSFA1b, but are scored as binding to the red node TFs. An interactive version of this network is available as an interactive file (Supplementary Cytoscape File S1).

**Fig. 6.**
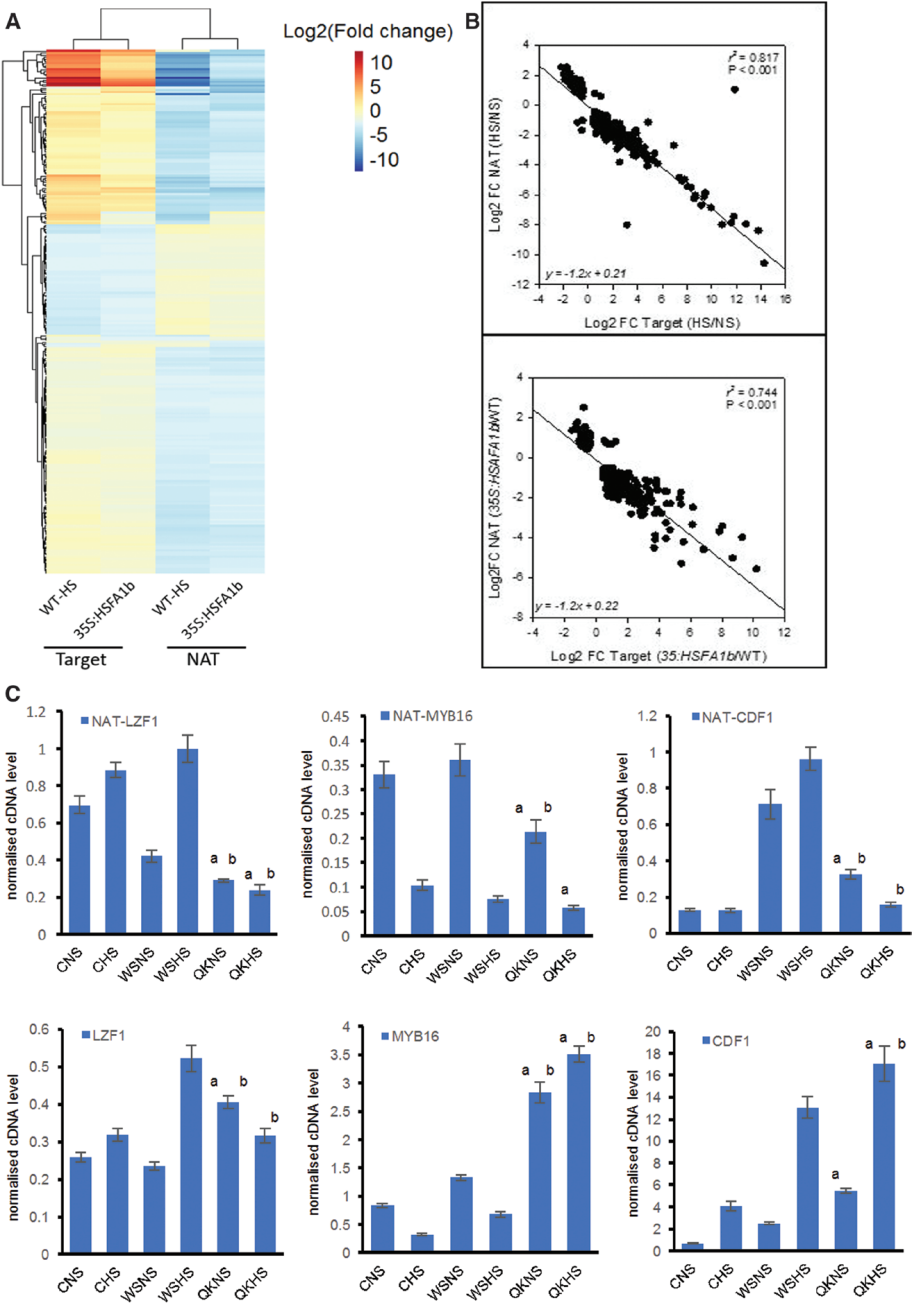
HSFA1b regulates expression of *cis*NAT genes and their target sense genes. (A) Heat map showing differentially expressed *cis*NAT genes and their putative sense targets in WT plants in HS compared with NS and in 35S:HSFA1b NS plants compared with WT NS plants. (B) Linear correlation plots showing the relationship between *cis*NAT and sense target transcript abundance for WT plants subjected to HS compared with NS (top panel) and for 35S:HSFA1b NS plants compared with WT NS plants (bottom panel). (C) Transcript levels determined by qPCR of selected *cis*NAT genes and their TF gene sense target in HS and NS qKO plants compared with their parental genotypes as in the legend of Fig. 5B. The suffixes ‘a’ and ‘b’ are where the qKO mutant shows a significant difference (*P*<0.05) under the same conditions (NS or HS) from Col-0 (C) and Ws-0 (W) respectively.

By intersecting the ChIP-seq data from NP:HSFA1b plants under HS and NS conditions (Supplementary Data S1) with the DEGs from NS 35S:HSFA1b plants compared with NS WT plants (Supplementary Data S3), we classified 1821 genes as differentially expressed in WT HS plants and in 35S:HSFA1b NS plants that were not bound by HSFA1b (Supplementary Data S1, S3). These were designated as indirectly regulated by *HSFA1b*, of which 281 are associated with development (Supplementary Data S7). We reasoned that this indirect regulation is achieved by *HSFA1b* being able directly to control the expression of other transcription regulatory genes, such as TF genes. We identified 27 TF genes as directly regulated by *HSFA1b* (Fig. 5A; Supplementary Data S1, S3). Eight of these genes have effects on growth and development (Fig. 5A; Kuno *et al.*, 2003; Achard *et al.*, 2008; Kolmos *et al.*, 2014; Wunderlich *et al.*, 2014; Kang *et al.*, 2015; Xu *et al.*, 2015; Valenzuela *et al.*, 2016). A selection of seven development-associated TF genes directly regulated by HSFA1b were tested for altered expression in NS and HS qKO plants compared with their parental genotypes (Liu *et al.*, 2011). In all examples, the expression of these genes was down-regulated in qKO plants compared with at least one parental genotype under NS and/or HS conditions (Fig. 5B), confirming their regulation by clade A1 HSFs (see also Fig. 6C).

*HSFA1b* and eight of its direct TF gene targets, *BASIC LEUCINE ZIPPER28* (*BZIP28*), *REVIELLE7* (*RVE7*), *SALT INDUCED ZINC FINGER1* (*SZF1*), *HSFB2b*, *G-BOX BINDING FACTOR3* (*GBF3*), *HSFB2a*, *RVE1*, and *ETHYLENE RESPONSE FACTOR13* (*ERF13*; Fig. 5A) are represented in the Arabidopsis Cistrome Atlas (O’Malley *et al.*, 2016). The Cistrome Atlas is a database of genome-wide TF-binding sites experimentally determined by DNA affinity purification sequencing (DAP-seq) (O’Malley *et al.*, 2016). Cistrome-generated binding data for HSFA1b showed a highly significant overlap, capturing 48% of the sites determined by ChIP-seq (Fig. 5C). To determine if the eight directly HSFA1b-regulated TFs connect to further TF genes, all differentially expressed TF gene targets of these eight TFs were collected. These were classified into those connecting to at least one of the eight directly HSFA1b-regulated TF genes, those also targeted directly by HSFA1b, and, finally, those also connecting to other indirectly *HSFA1b*-regulated TF genes in the data set. The resulting hierarchical network of direct interactions between TF genes consists of 183 nodes and 546 edges, the top node being HSFA1b because it has no inward connections (Fig. 5D). The network model visually demonstrates that HSFA1b is capable of indirectly regulating gene expression by acting upon the expression of other TF genes at least one or two steps removed from a direct interaction with it. While this paper was being written, a limited ChIP-seq data set became available for BZIP28 (Zhang *et al.*, 2017). Of the 133 BZIP28 target genes in seedlings subject to tunicamycin treatment (see the Discussion), 29 were differentially expressed in 35S:HSFA1b NS leaves, resulting in a simple illustrative network of indirect regulation of the transcript levels of these genes by HSFA1b via BZIP28 (Supplementary Fig. S6 at *JXB* online.

Indirect regulation of gene expression by HSFA1b could also occur via its interactions with 817 *cis*NAT genes under NS and/or HS conditions (Table 1; Fig. 2D–F; Supplementary Data S1). RNA-seq data revealed that 413 of these *cis*NAT genes were differentially expressed in HS WT plants and/or 35S:HSFA1b NS plants (Fig. 6A). These NAT transcripts correspond to 357 putative sense target loci (Supplementary Data S8). GO classification of the *cis*NAT target genes revealed enrichment only for stress-associated functions (Supplementary Data S8). However, the only significantly enriched GO Molecular Function class was for 39 gene targets coding for transcription regulators [false discovery rate (FDR) ≤0.1; Supplementary Data S8]. Of these, eight [*LIGHT REGULATED ZINC FINGER PROTEIN1* (*LZF1*), *RELATED TO AP2.7* (*RAP2.7*), *ARABIDOPSIS NAC DOMAIN CONTAINING PROTEIN56* (*ANAC056*), *ANAC078*, *ANAC083*, *HOMEOBOX PROTEIN2* (*HB2*), *MYB DOMAIN CONTAINING PROTEIN16* (*MYB16*),and *CYCLING DOF FACTOR1* (*CDF1*)] have development-associated functions (Supplementary Table S1). There was a high negative correlation between the differential levels of HS- and 35S HSFA1b-responsive NAT RNAs and the transcript levels of their overlapping sense targets (Fig. 6A, B). Reciprocal differential levels between at least one WT genotype and qKO of selected NAT antisense and their sense cognate transcripts under NS and/or HS were also observed (Fig. 6C).

### HSFA1b is one of eight TFs that regulate a common set of stress and developmental genes

**Fig. 7.**
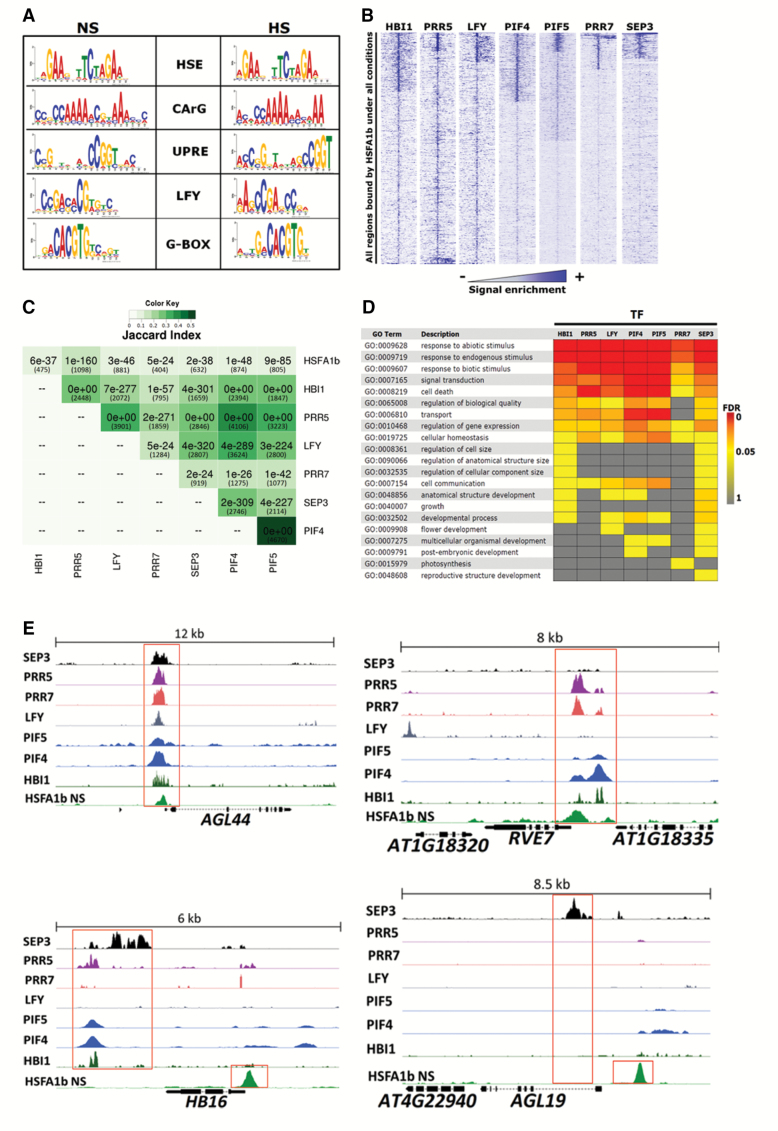
Seven TFs co-ordinate with HSFA1b. (A) All significant motifs within HSFA1b peaks discovered by MEME (*P*<0.0001) in the NS and HS data sets. (B) Density heat maps showing enrichment of ChIP-seq signals of the seven TFs on 10 kb around the regions bound by HSFA1b. (C) Combined Jaccard index and pHYPER correlation matrix showing the significance of overlaps between the target genes of the seven TFs and HSFA1b under NS conditions only. Numbers of genes in each overlap are in parentheses. (D) GO Slim analysis comparing the enriched Biological Proess terms of the common targets between HSFA1b and the other seven TFs. (E) Genome browser view of normalized ChIP-seq tags from the ChIP-seq data of the eight TFs showing examples of target genes bound by HSFA1b and up to seven other TFs.

The TF networks depicted (Fig. 5D; Supplementary Fig. S6) do not consider any convergence of signalling from other networks. To determine how HSFA1b could co-operate with other TFs, we searched for enriched TF-binding motifs present within the HSFA1b ChIP-seq peaks (see the Materials and methods). As expected, a consensus heat shock *cis*-element (HSE) was identified from these enriched peak sequences (Fig. 7A). Reported variant HSEs, such as gapped HSEs (Guo *et al.*, 2008) and HSE1b motifs (Bechtold *et al.*, 2013, were not detected. Likewise, TC-rich elements and STRE motifs bound by HSFA1a *in vitro* (Guo *et al.*, 2008) were absent from the data set. In addition, four significantly over-represented conserved known motifs were identified in both the NS and HS data sets (Fig. 7A). These were the G-box motif, recognized by various TFs, including BZIP, BASIC HELIX LOOP HELIX (BHLH), and PSEUDO RESPONSE REGULATOR (PRR) TFs (Chawade *et al.*, 2007; Nakamichi *et al.*, 2012), the CArG element bound by MADS-box TFs (Moyroud *et al.*, 2011), the recognition motif of the LEAFY (LFY) TF (Pajoro *et al.*, 2014), and the Unfolded Protein Responsive Element (UPRE) (Martínez and Crispeels, 2003).

Published ChIP-seq data from seven TFs known to bind to the co-occurring motifs significantly overlapped with HSFA1b target regions under NS and HS (Fig. 7B, Supplementary Data S9; see the Materials and methods). The TFs were HOMOLOG OF BEE2 INTERACTING WITH IBH1 (HBI1; a BHLH TF; Fan *et al.*, 2014), LFY (Pajoro *et al.*, 2014), SEPALLATA3 (SEP3; a MADS-box TF; Pajoro *et al.*, 2014), PHYTOCHROME INTERACTING FACTOR4 (PIF4) and PIF5 (BHLH TFs; Pedmale *et al.*, 2016), PRR5, and PRR7. Furthermore, there was considerable overlap in target genes not only between HSFA1b and the seven TFs but also between the seven TFs (Fig. 7C; Supplementary Data S9), examples of which are shown in Fig. 7E. Since the published ChIP-seq data sets were from NS plants, we scored for co-occurrence of the seven TF motifs only in Groups I and II (i.e. under NS conditions; Supplementary Data S9). In Group I genomic features, shared sites were between 3% and 6.4% and in Group II between 15.5% and 27.8%.

The target genes shared between HSFA1b and each of the seven TFs (Supplementary Data S9) showed significant enrichment of GO terms for response to stress and endogenous stimuli (Fig. 7D; Supplementary Data S10). Also, apart from the PRR TFs, there was significant enrichment of GO terms for developmental processes (Fig. 7D; Supplementary Data S10). The expression of *PRR5*, *HBI1*, *PRR7*, and *PIF5* was responsive to HS (*q*=0.0003) but not to *HSFA1b* overexpression (Supplementary Data S3), while *PIF4*, *LFY*, and *SEP3* were responsive to neither HS nor overexpression of *HSFA1b*. There are 76 targets for all eight TFs, of which 15 are associated with developmental processes (Table 2). Thirty-nine of these (including 10 developmental genes) responded to HS, and 18 (including two developmental genes) showed significant changes in transcript abundance in response to *HSFA1b* overexpression (Table 2; Supplementary Data S3).

**Table 2.** Target genes common to HSFA1b (NS and HS), PRR5, PRR7, HBI1, LFY, SEP3, PIF4, and PIF5

Locus	Gene symbol	Locus	Gene symbol	Locus	Gene symbol
*AT1G05370*	AT1G05370	AT2G45820	AT2G45820	AT4G28230	AT4G28230
AT1G07580	AT1G07580	*AT2G45960*	PIP1B	AT4G37260	MYB73
AT1G13245	RTFL17	*AT3G10985*	SAG20	AT4G39838	AT4G39838
**AT1G13250**	GATL3	AT3G11415	AT3G11415	**AT5G01600**	FER1
*AT1G14920*	GAI	AT3G11700	FLA18	AT5G08139	AT5G08139
AT1G17990	AT1G17990	*AT3G14440*	NCED3	***AT5G11740***	AGP15
*AT1G18740*	AT1G18740	AT3G15200	AT3G15200	***AT5G13100***	AT5G13100
*AT1G21380*	AT1G21380	*AT3G15210*	ERF4	***AT5G21940***	AT5G21940
***AT1G29640***	AT1G29640	***AT3G15770***	AT3G15770	*AT5G22940*	F8H
***AT1G32640***	MYC2	AT3G15790	MBD11	**AT5G24530**	DMR6
***AT1G32920***	AT1G32920	*AT3G16240*	DELTA-TIP	AT5G25220	KNAT3
**AT1G72450**	JAZ6	AT3G22380	TIC	***AT5G47220***	ERF2
**AT1G77280**	AT1G77280	AT3G24518	AT3G24518	AT5G47225	AT5G47225
*AT1G78070*	AT1G78070	*AT3G24520*	HSFC1	*AT5G48250*	BBX8
AT1G80440	AT1G80440	***AT3G49790***	AT3G49790	***AT5G49520***	WRKY48
AT2G14210	AGL44	AT3G50750	BEH1	*AT5G53400*	BOB1
AT2G22426	AT2G22426	*AT3G59060*	PIL6	*AT5G57660*	COL5
*AT2G23290*	MYB70	AT3G59940	AT3G59940	***AT5G58070***	TIL
*AT2G23430*	ICK1	AT4G00360	CYP86A2	AT5G60680	AT5G60680
*AT2G28550*	RAP2.7	*AT4G01250*	WRKY22	AT5G61970	AT5G61970
**AT2G29660**	AT2G29660	AT4G01720	WRKY47	AT5G62000	ARF2
AT2G41890	AT2G41890	*AT4G23630*	BTI1	***AT5G62430***	CDF1
AT2G41900	OXS2	*AT4G26700*	FIM1	AT5G65305	AT5G65305
*AT2G41940*	ZFP8	*AT4G27260*	WES1	*AT5G67300*	MYBR1
AT2G44810	DAD1	AT4G27510	AT4G27510	*AT5G67420*	LBD37
AT2G45660	AGL20				

Summary of genes responsive to *HSFA1b* overexpression under NS and HS (bold) and HS in the WT (italics) are from Supplementary Data S3 (*q*≤0.05). Underlined loci are developmental genes.

## Discussion

### *HSFA1b* regulates growth- and development-associated genes as well as stress resistance genes

Genome-wide binding and transcript profiling has shown how *HSFA1b* directly and indirectly regulates the expression of genes coding for resistance to abiotic and biotic stress (Figs 1C, 3C, 4C; Supplementary Data S2, S5, S6). This is consistent with previous studies and confirms that both WT and *HSFA1b*-overexpressing plants in this study responded typically to this moderate HS treatment (Prändl *et al.*, 1998; Guo *et al.*, 2008; Chauhan *et al.*, 2011; Nishizawa-Yokoi *et al.*, 2011; Bechtold *et al.*, 2013; Jung *et al.*, 2013; Guo *et al.*, 2016; Jacob *et al.*, 2017).

HSFA1b also targets and influences the expression of up to 354 genes involved in plant growth and development (Figs 1C, 3C, 4C; Supplementary Data S2, S5, S6; Supplementary Table S1). The molecular functions of these genes are very diverse (Figs 1C, 3C, 4C, 5A; Table 2; Supplementary Table S1; Supplementary Data S2, S5–S7; S10). These range from cell integrity-associated chaperones engaged in chloroplast development, hormone metabolism (auxins and brassinosteroids), photoreceptors, components of photomorphogenesis signalling, cell wall synthesis enzymes, development-associated TFs, and to the defective alleles of genes associated with growth and development phenotypes (Supplementary Table S1). Therefore, we could not identify a discrete group of genes that would explain the phenotypic effects of *HSFA1b* overexpression or the effect of a moderate HS on growth of WT plants. Instead, our data show that many genes of diverse function are affected. We suggest that the wide-ranging but subtle effects that moderate HS and *HSFA1b* overexpression has on plant growth (Supplementary Figs S3, S5A–D; Bechtold *et al.*, 2013) is the net consequence of this effect upon widely diverse cellular functions.

### Reconfiguration of HSFA1b genome-wide binding going from NS to HS

There was a clear difference in HSFA1b binding profiles to genomic regions under HS and NS conditions such that three groups (I–III) could be distinguished (Figs 1A, B, D, E; Supplementary Data S1). Therefore, within the first 30 min of HS, HSFA1b ceases to engage with 124 of these genes (Group I) that are predominantly associated with growth and development functions and targets 553 protein-coding genes (Group III) of which a substantial number are associated with defence against environmental stress (Supplementary Data S2). Group II HSFA1b target genes, bound under both conditions, have enrichment for GO terms in both stress responses and growth and development (Supplementary Data S2). It has been previously shown that there is a substantial genome-wide shift in the distribution of open chromatin in transitioning from NS to HS, which changes accessibility of HSEs (Sullivan *et al.*, 2014). This suggests that this re-configuration of HSFA1b binding targets is associated with a distinct distribution of genome-wide DNase I-hypersensitive sites (Supplementary Fig. S4).

### The indirect regulation of gene expression by *HSFA1b* through a network of TF genes

Of the 2121 genes that show differential transcript levels in HS-exposed WT and 35S:HSFA1b plants under NS, 84% were not targets for HSFA1b binding (Supplementary Data S1, S3). Such genes were classified as indirectly regulated by HSFA1b and include 281 development-associated genes (Supplementary Data S6, S7). Therefore, *HSFA1b* could exert its influence over stress defence and growth-associated processes through an extensive transcriptional regulatory network. We depicted this network as hierarchical because *HSFA1b* transcript levels do not vary substantially in response to environmental stress (Nishizawa *et al.*, 2006; Swindell *et al.*, 2007; Bechtold *et al.*, 2013; Sullivan *et al.*, 2014). This is in contrast to the transcriptional regulation of all the other TF genes considered here. We identified 27 TF genes that were direct targets of HSFA1b that showed differential expression in response to HS in WT plants, overexpression of *HSFA1b*, and, for a sample of seven of them, altered expression in the qKO mutant (Fig. 5A, B). The regulation by HSFA1b of the expression of these many TF genes implies considerable complexity even in a network only one step removed from direct regulation and shows how large such networks could be (Fig. 5D; Supplementary Cytoscape File S1). However, the TF binding data from the Cistrome Atlas used to generate the network overestimates the number of binding events that would occur *in vivo*, which is the case for HSFA1b (Fig. 5C). Furthermore, for some TF families this assay does not work (O’Malley *et al.*, 2016). Nevertheless, despite these limitations, the resulting network clearly depicts the potential for layers of indirect regulation of gene expression by HSFA1b. The recent availability of a ChIP-seq data set for BZIP28 (Fig. 5A; Zhang *et al.*, 2017) from seedlings undergoing a tunicamycin-induced unfolded protein response (UPR) confirmed this notion of indirect regulation by HSFA1b of other TF genes (Supplementary Fig. S6).

A potentially confounding factor in the classification of direct and indirect regulation of gene expression by *HSFA1b* is the reliance upon 35S:HSFA1b plants under NS conditions. However, the 50-fold *HSFA1b* overexpression in the 35S:HSFA1b line chosen (Supplementary Data S3; Supplementary Fig. S1; Bechtold *et al.*, 2013) did not alter transcript levels over and above the levels encountered in WT plants subject to HS (Fig. 4A). Nevertheless, 28% of DEGs in the 35S:HSFA1b plants were not responsive to HS (see the Results and Supplementary Data S3). This is to be expected since *HSFA1b* does control responses to stresses other than HS. These include resistance to infection by *Pseudomonas syringae* and the oomycete *Hyaloperonospora arabidopsidis*, oxidative stress, high light stress, and drought stress (Bechtold *et al.*, 2013; Jung *et al.*, 2013). Therefore, not all genes whose expression is altered in 35S:HSFA1b plants would necessarily be expected to respond to HS in WT plants. However, we cannot rule out that some genes are aberrantly expressed because of high *HSFA1b* overexpression levels, although some surety was also provided by confirming altered responses of selected genes in the qKO mutants (Fig. 5B; see the Results).

### *HSFA1b* control of *cis*NAT gene expression

Genome-wide binding of HSFA1b showed a preference for binding internal to or downstream of the TSS of protein-coding genomic loci under NS conditions and less so under HS (Fig. 1D, E). This was associated with HSFA1b targeting 817 *cis*NAT RNA genes and 79 lincRNA genes (Supplementary Data S1), and 51% of these were differentially expressed under HS and/or in 35S:HSFA1b plants (Table 1; Supplementary Data S8). In general, lincRNA levels are differentially regulated in response to abiotic and biotic stress, and *cis*NAT RNAs form 10–30% of the total non-coding RNA complement (Liu *et al.*, 2012, 2015*a*, *b*; Yu *et al.*, 2013; Wang *et al.*, 2014; Ariel *et al.*, 2015; Bouchard *et al.*, 2015; Muthusamy *et al.*, 2015; Shafiq *et al.*, 2016). In contrast, the fact that *HSFA1b* regulates the expression of many more *cis*NAT genes than lincRNA genes suggests that it specifically targets them for regulation (Table 1; Supplementary Data S1, S8). This greatly extends observations made on the regulation of *HSFB2a* and its *asHSFB2a cis*NAT gene, which show reciprocal transcript levels (Wunderlich *et al.*, 2014) and is termed discordant expression (Wang *et al.*, 2014). From the transcriptomics analysis of the 412 HS- and HSFA1b-regulated *cis*NATs, >98% of them and their partner sense transcript showed discordant expression (Fig. 6A, B; Supplementary Data S8), contrasting with de-etiolating seedlings where ~55% showed this pattern (Wang *et al.*, 2014). Indirect regulation by *HSFA1b* of gene expression could occur via its direct regulation of *cis*NAT RNA levels and the eventual silencing (si)RNAs generated from them. However, in de-etiolating Arabidopsis seedlings, siRNAs play no role in the light regulation of *cis*NAT–target gene pairs (Wang *et al.*, 2014). Instead, a correlation was noted between *cis*NAT gene expression and histone H3 acetylation in dark and light conditions. Acetylation of histones is mediated by non-coding RNAs (Groen and Morris, 2013; Wang *et al.*, 2014) and, in animals, is co-ordinated with that of HSFs (Erkina and Erkine, 2006; Petesch and Lis, 2008; Akerfelt *et al.*, 2010; Guertin and Lis, 2010). We speculate that the same could happen in plant cells undergoing a transition from NS to HS and could be how co-ordinated changes in chromatin condensation and HSFA1b binding occur (Supplementary Fig. S4). In addition, *cis*NATs have been shown to enhance translation of the target mRNA (Jabnoune *et al.*, 2013; Bazin *et al.*, 2017). Therefore, the HSFA1b-mediated control of *cis*NAT gene expression may lead to altered translation of specific transcripts under HS conditions.

### HSFA1b is one route for the transmission of environmental cues to a core set of stress-responsive and development-associated genes

In Arabidopsis, genomic regions are occupied by multiple TFs and enriched for genes involved in development and stimulus responses (Heyndrickx *et al.*, 2014). The notion of co-operation with other TFs is supported by the clear enrichment of co-occurring binding motifs in promoter regions of HSFA1b-bound genes under NS and HS conditions (Fig. 7A). In the ChIP-seq data sets available to us from NS plants, we compared them with our NP:HSFA1b NS data only. This revealed 76 gene targets common to the eight TFs (Fig. 7B, C, E; Supplementary Data S9). These eight TF genes have all been implicated in the control of growth and development and responses to environmental stress. For example, HBI1 is important in poising plants between growth and the level of immunity to pathogens (Fan *et al.*, 2014). PRR5, PRR7, PIF4, PIF5, LFY, and SEP3 regulate genes responsive to cold stress, oxidative stress, light quality, and photoperiod, as well as playing developmental roles (Moyroud *et al.*, 2011; Nakamichi *et al.*, 2012; Pajoro *et al.*, 2014; Pedmale *et al.*, 2016). We suggest that HSFA1b is one of at least eight and probably many more TFs that transduce a variety of endogenous and environmental signals controlling different combinations of genes drawn from a core group of development- and stress-associated genes that control the plant’s multiple physiological responses to the highly variable environment it encounters.

## Supplementary data

Supplementary data are available at *JXB* online.

Methodology. A detailed step-by-step protocol for the preparation of ChIP-seq samples from Arabidopsis leaves.

Fig. S1. Properties of the HSFA1b–eYFP line used for ChIP-seq.

Fig. S2. Time series qRT-PCR results comparing the activation time of four heat-responsive genes.

Fig. S3. The impact of heat stress on growth of Arabidopsis shoots.

Fig. S4. The degree of overlap between Groups I–III HSFA1b target genes and the nearest transcription start site (TSS) loci in genome-mapped DNase I-hypersensitive sites.

Fig. S5. Phenotype of *35S:HSFA1b* plants compared with Col-0.

Fig. S6. Hierarchical network showing interaction between HSFA1b and BZIP28.

Table S1. Experimentally confirmed developmental genes bound by HSFA1b

Table S2. Primers used in the qPCR analyses.

Data S1. All genomic sites bound by HSFA1b under NS and HS

Data S2. Significant GO terms of HSFA1b target genes in Groups I, II, and III.

Data S3. All differentially expressed genes in WT HS versus WT NS, 35S:HSFA1b versus WT NS, and 35S:HSFA1b versus WT HS.

Data S4. Expression of all Group I, II, and III genes in WT HS compared with WT NS.

Data S5. All significant GO terms of up-regulated and down-regulated Group I, II, and III genes in response to HS.

Data S6. All significant GO terms of up- and down-regulated Group I, II, and III genes in 35S:HSFA1b NS.

Data S7. Genes whose expression is indirectly regulated by HSFA1b.

Data S8. Target genomic loci and their *cis*NAT RNAs that show differential expression.

Data S9. Shared binding sites between HSFA1b and HBI-1, PRR5, LFY, SEP3, PIF4, PIF5, and PRR7.

Data S10. All significantly enriched GO terms of the genes targeted by HSFA1b and HBI-1, LFY, PRR5, PRR7, SEP3, PIF4, and PIF5.

Cytoscape File S1. A Cytoscape file which allows an interactive view of Fig. 5D but requires first that the open source program is downloaded from http://www.cytoscape.org/.

Supplementary Figures and Tables

Supplementary Dataset S1-S9

Supplementary Document
