# Supplementary material for: Arabidopsis HEAT SHOCK TRANSCRIPTION FACTORA1b regulates multiple developmental genes under benign and stress conditions
Source: J Exp Bot. 2018 Apr 25;69(11):2847–62. doi: 10.1093/jxb/ery142 (PMC5961379; doi:10.1093/jxb/ery142)
Supplement: Supplementary Figures and Tables [file ery142_suppl_supplementary_figures_s1-s6_tables_s1-s2.pdf]

Supplementary Data

Arabidopsis *Heat Shock Transcription FactorA1b* regulates multiple developmental genes under growth and stress conditions

Waleed S. Albihlal[1], Igor Chernukhin[2], Thomas Blein[3], Ramona Persad, Irabonosi Obomighie, Martin Crespi[3], Ulrike Bechtold and Philip M. Mullineaux*

Supplementary Methodology

Chromatin immunoprecipitation followed by high throughput sequencing Ch.

This protocol was optimized to perform ChIP-seq on Arabidopsis leaves only. Further optimization may be required to conduct this experiment on other tissue.

This protocol is adapted and modified from Saleh et al. (2008) from where buffer and solution formulations can be found. The protocol adapted from this published procedure was as follows:

1- 2 g of 5-week-old Arabidopsis leaves were harvested and placed in a 100 ml beaker.
2- 40 ml of crosslinking buffer was added to each sample followed by applying a vacuum for 10 minutes at room temperature.
3- The crosslinking reaction was quenched by adding 2.5 ml of 2 M Glycine (final concentration 125 mM), mixed well and followed by applying a vacuum for an additional 5 minutes at room temperature.
4- Plant tissue was washed three times with sterile water; water was removed from the plant material by blotting the plant tissue between paper towels then leaves were snap frozen in liquid nitrogen.
5- Frozen plant tissue was ground to very fine powder using a dry-ice pre-chilled mortar and pestle.
6- Ground samples were placed in pre-chilled 50 ml falcon tubes and kept on dry ice.
7- Ground plant material was re-suspended in 25 ml of cold nuclei isolation buffer, vortexed briefly to mix and kept on ice for 15 to 20 minutes with repeated vortexing every 5 minutes until complete homogenization was achieved and no visible clumps remain.
8- The homogenized slurry was filtered through four layers of cheesecloth (Fisher scientific, S04824). A funnel was placed on a new 50 ml falcon tube then the four layers of cheesecloth were placed on the funnel. Homogenized slurry was poured on the cheesecloth then cheesecloths were manually squeezed without allowing any solid material to pass into the collection tube.
9- Filtrates were centrifuged at 4500 x g for 30 minutes at 4°C until a tight white pellet (nuclei) with an overlay of chlorophyll was visible.

10- The supernatant was discarded and pellet (nuclei) were re-suspended in 2 ml of cold nuclei lysis buffer.
11- Samples were divided into four aliquots of 500 µl each in 1.5 ml eppendorf tubes.
12- Chromatin was sheared to an average fragment size of ~250bp (100bp to 350bp) by sonication. The sonicator used was a Bioruptor diagenode standard. The sonication conditions were 4 cycles, each cycle was 10 minutes divided into 30 seconds of sonication and 30 seconds pause at the highest power setting. Samples were kept on ice for at least 2 minutes between each cycle to avoid heating and foaming of the samples.
13- Samples were centrifuged at 13000 x g for 10 minutes at 4°C to pellet debris.
14- The supernatants were transferred into new 1.5 ml microfuge tube.
15- Sonicated and non-sonicated chromatin were reverse-crosslinked (see step 26) and DNA was extracted using phenol/chloroform method (see steps 27 – 36) followed by subjecting them to electrophoresis side by side through a 1.5% (w/v) agarose gel to compare the size distribution of sonicated and non-sonicated chromatin.
16- 300 µl aliquot was transferred into new 1.5 ml tube and diluted with 700 µl of lysis buffer.
17- 5 µl of the Anti-YFP antibody (Abcam, ab290) was added to the diluted chromatin, making a final dilution of 1:200 and incubated for 3 hours at 4°C with gentle rotation.
18- 60 µl of pre-equilibrated and pre-blocked Protein A-Sepharose CL4-B beads (Sigma-Aldrich, P3391) were added sample followed by further incubation for 2 hours at 4°C with gentle rotation.
19- Samples were centrifuged at 2000 x g for 5 minutes at 4°C. The beads-antibody-chromatin complexes were recovered as pellets.
20- The beads were washed three times with 1 ml of low salt buffer for 5 minutes with gentle rotation. Then three times with 1 ml high salt buffer and finally once with lithium chloride wash buffer.
21- Immune-complexes were eluted from beads by adding 250 µl of freshly prepared elution buffer (see **reagent formulations** below) followed by incubation at room temperature for 15 minutes with gentle rotation.
22- Samples were centrifuged at 2000 x g for 5 minutes at 4°C and supernatant was transferred into new 2 ml microfuge tube.
23- The elution step 21 was repeated by adding another 250 µl of freshly prepared elution buffer to the beads followed by incubation at room temperature for 30 minutes with gentle rotation.
24- Samples were centrifuged at 2000 x g for 5 minutes at 4°C and the supernatant was collected.
25- The two eluates (from steps 21 and 23) were combined to a total eluate of 500 µl.
26- 20 µl of 5 M NaCl was added to each sample followed by a 12 hour incubation at 65°C to reverse the crosslinking.

27- The following solutions were added to samples the following day: 10 μl of 0.5 M EDTA, 20 μl of 1 M Tris-HCl, and 1 μl of proteinase K (20 mg ml^{-1}; ThermoFisher, EO0491) followed by incubation for 1.5 h at 42°C to digest the proteins.
28- 1 volume of water saturated phenol pH 7.9 (FisherScientific, BP1750I-400) was added to samples followed by vortexing for 10 seconds.
29- Samples were centrifuged at 13000 x g for 15 minutes at 4°C and the upper phase (~500 μl) was transferred into a new 2 ml microfuge tube.
30- 200 μl of water-saturated chloroform was added to samples followed by vortexing for 10 seconds.
31- Samples were centrifuged at 13000 x g for 15 minutes at 4°C.
32- The upper phase of each sample was transferred into 2 ml microfuge tubes.
33- The following was added to the transferred samples: 2.5 volume of absolute ethanol, 0.1 volume of 3 M sodium acetate pH 5.2, 3 μl of glycogen (20 mg ml^{-1}) followed by incubation for 2 hours at -80°C.
34- Samples were centrifuged at 13000 x g for 30 minutes at 4°C.
35- Supernatants were discarded and pellets washed with 500 μl of 75% (v/v) ethanol. Pellets were air dried at room temperature for 7 minutes.
36- Pellets were then dissolve in 40 μl of sterile water.
37- The control samples were wild type plants treated exactly the same way as the test samples.
38- Sequencing libraries were constructed using TruSeq ChIP library preparation kit (Illumina, IP-202-1012) at Earlham institute (formerly known as The Genome Analysis Centre, Norwich, UK).
39- Samples were multiplexed then sequenced by the Earlham Institute on Illumina HiSeq2000 platform with the following metrics: 100 bp paired-end and a minimum depth of 10 million reads per library.

Notes:

1- The choice of antibody is very crucial of for the success of ChIP. The antibody must be a ChIP or co-IP grade. Also, polyclonal antibodies are preferred over monoclonal antibodies for ChIP experiments. If no ChIP or co-IP antibodies are available then it is recommended to test the antibodies on crosslinked materials by performing IP followed by western blot.
2- The choice of beads is also critical for the success of any immunoprecipitation experiment. It is very important to know whether Protein A or Protein G has the highest affinity for the antibody used in the experiment.
3- Crosslinking is a key step for the success of ChIP. Adjustments have to be done if more than 2 g of plant material is to be used. The volumes of the crosslinking buffer and the glycine solution have to be increased accordingly. Cramming too much plant material in 40 ml of crosslinking buffer will result in a very weak or no crosslinking.

Reagent formulations:

1- **Crosslinking buffer:**
0.4 M sucrose, 10 mM Tris-HCl pH 8.0, 1 mM EDTA, 1% formaldehyde, and 1 mM PMSF.

Note: Use high quality formaldehyde and make fresh buffer before each experiment. The presence of sucrose in the crosslinking buffer increases the efficiency of DNA-protein crosslinking. The protease inhibitor (PMSF) should be added directly before using the buffer as PMSF has a very short life in aqueous solutions.

2- **Nuclei Isolation buffer:**
0.25 M sucrose, 15 mM PIPES pH 6.8, 5 mM MgCl2, 60 mM KCl, 15 mM NaCl, 1 mM CaCl2, 0.9% Triton X100, 1 mM PMSF, 2 μg ml^{-1} Pepstatin A, and 2 μg ml^{-1} Aprotinin.

Note: Prepare fresh, filter sterilize and keep at 4°C. The protease inhibitors PMSF, Pepstatin A and Aprotinin should be added directly before using the buffer.

3- **Nuclei Lysis buffer:**
50 mM HEPES pH 7.5, 150 mM NaCl, 1 mM EDTA, 0.1% SDS, 0.1% sodium deoxycholate, 1% Triton X100, 1 mM PMSF, 1 μg ml^{-1} Pepstatin A, and 1 μg ml^{-1} Aprotinin.

Note: Prepare fresh, filter sterilize and keep at 4°C. The protease inhibitors PMSF, Pepstatin A and Aprotinin must be added immediately before using the buffer.

4- **Low Salt wash buffer:**
150 mM NaCl, 20 mM Tris-HCl pH 8.0, 2 mM EDTA, 0.2% SDS, and 0.5% Triton X100, 1 mM PMSF, 2 μg ml^{-1} Pepstatin A, and 2 μg ml^{-1} Aprotinin.

5- **High Salt wash buffer:**
500 mM NaCl, 20 mM Tris-HCl pH 8.0, 2 mM EDTA, 0.2% SDS, and 0.5% Triton X100, 1 mM PMSF, 2 μg ml^{-1} Pepstatin A, and 2 μg ml^{-1} Aprotinin.

6- **Lithium Chloride wash buffer:**
0.25 M lithium chloride, 1% sodium deoxycholate, 10 mM Tris-HCl pH 8.0, 1 mM EDTA, 1% NP-40 1 mM PMSF, 2 μg ml^{-1} Pepstatin A, and 2 μg ml^{-1} Aprotinin.

7- **TE buffer:**
1 mM EDTA and 10 mM Tris-HCl pH8.0 1 mM PMSF, 2 μg ml^{-1} Pepstatin A, and 2 μg ml^{-1} Aprotinin.

8- **Elution buffer:**
0.5% SDS and 0.1 M sodium bicarbonate (NaHCO3).

Note: prepare fresh and keep at room temperature to avoid SDS precipitation.

9- **PMSF stock solution:**
Prepare a 100 mM stock solution in isopropanol or absolute ethanol. Aliquot and store at -20°C.

10- **Pepstatin A stock solution:**

Prepare a 1 mg ml^{-1} stock solution in methanol. Aliquot and store at -20°C.

11- **Aprotinin stock solution:**

Prepare a 1 mg ml^{-1} stock solution in sterile deionized water, aliquot and store at -20°C.

12- **Preparing Protein (A/G) sepharose beads (for ChIP-SEQ)**

a- Swelling and storage:

i- Resuspend protein (A/G) sepharose beads (250 mg) in 20 ml of sterile water for an overnight at 4°C. This will result in 1 ml swollen bead volume.

ii- Wash beads three time with 10 ml sterile water.

iii- Resuspend protein (A/G) sepharose beads in storage buffer (10 mM Tris-HCl pH 8.0, 1 mM EDTA, and 0.1% sodium azide.

iv- Make 50% slurry with storage buffer. Add enough volume of storage buffer so that the final volume of beads plus the storage buffer is twice the volume of beads alone. For example, if the sediment volume of beads is 1 ml then add enough storage buffer so that the total volume of beads plus storage buffer is 2 ml.

b- Pre-blocking beads:

i- Resuspend beads in 200 µl of pre-blocking buffer and mix with gentle rotation for 4 hours or an overnight at 4°C.

ii- Centrifuge the beads at 2000 x g for 5 minutes at 4°C and discard the supernatant.

iii- Pre-equilibrate beads with lysis buffer.

iv- Pre-blocking buffer:
10 µl glycogen (20 mg ml^{-1}), 10 µl BSA (20 mg ml^{-1}), and 20 µl yeast t-RNA (10 mg ml^{-1}) in 1 ml of lysis buffer plus protease inhibitors.

Notes:

- Pre-blocked beads can be used within 5 days.
- For long term storage, resuspend beads in storage buffer.

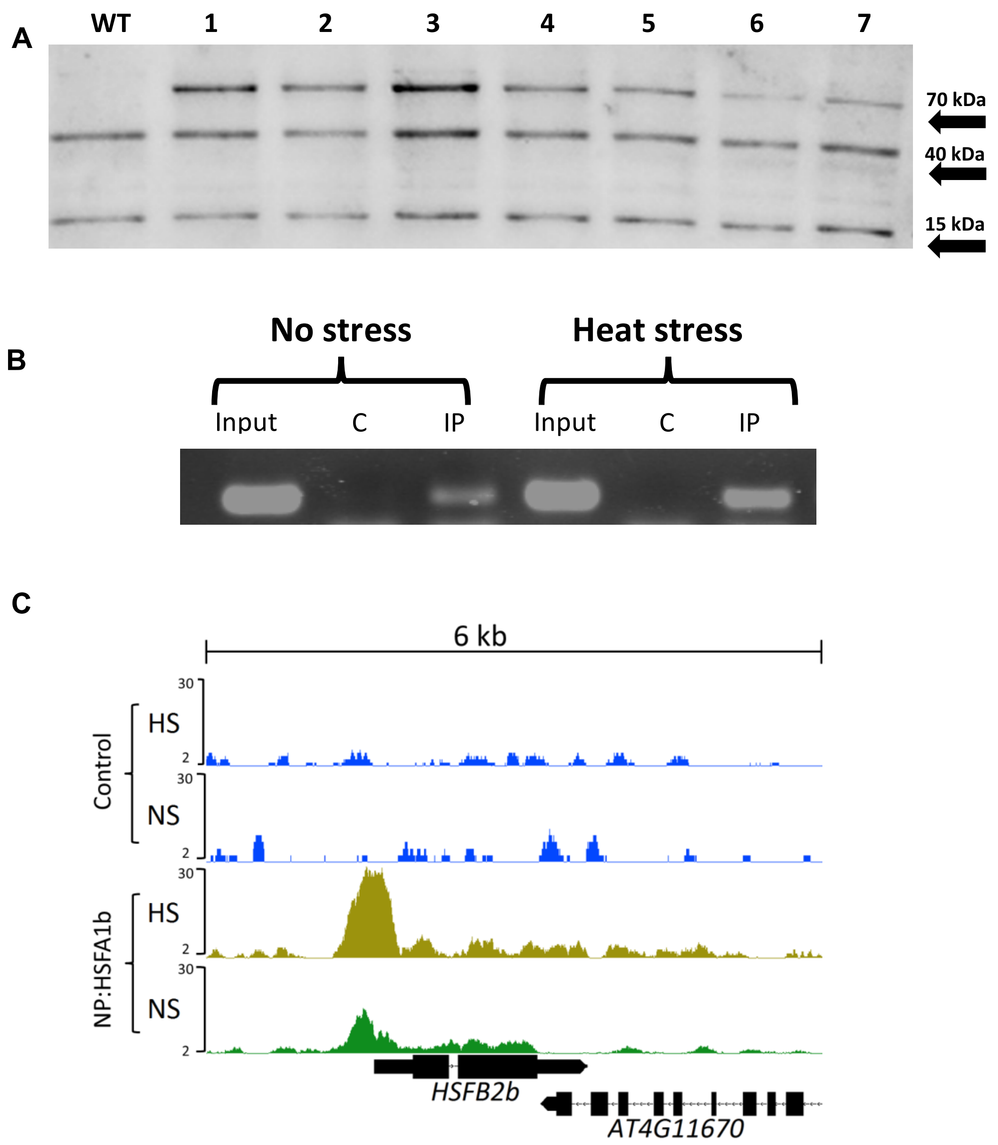

Figure S1 (A) Immunoblot comparing the levels of HSFA1b-eYFP extracted from seven NP:HSFA1b independent T3 transgenic lines. HSFA1b-eYFP was probed with the same GFP antibody used later in ChIP-PCR and ChIP-seq experiments. The HSFA1b-YFP fusion is the top band on the image. **(B)** PCR amplification of ChIP *HSFB2b* promoter fragments from transgenic line NP:HSFA1b_6 showing that this line is suitable for ChIP experiments under NS and HS. Input DNA is the positive control for the PCR reaction, C is a GFP antibody control precipitation with Col-0 (WT) and IP the NP:HSFA1b_6 sample that was immune-precipitated with GFP antibody. **(C)** Genome browser view of normalized NP:HSFA1b NS and NP:HSFA1b HS ChIP-seq tags on the promoter of *HSFB2b* confirming the ChIP-PCR result. Control is as in panel B

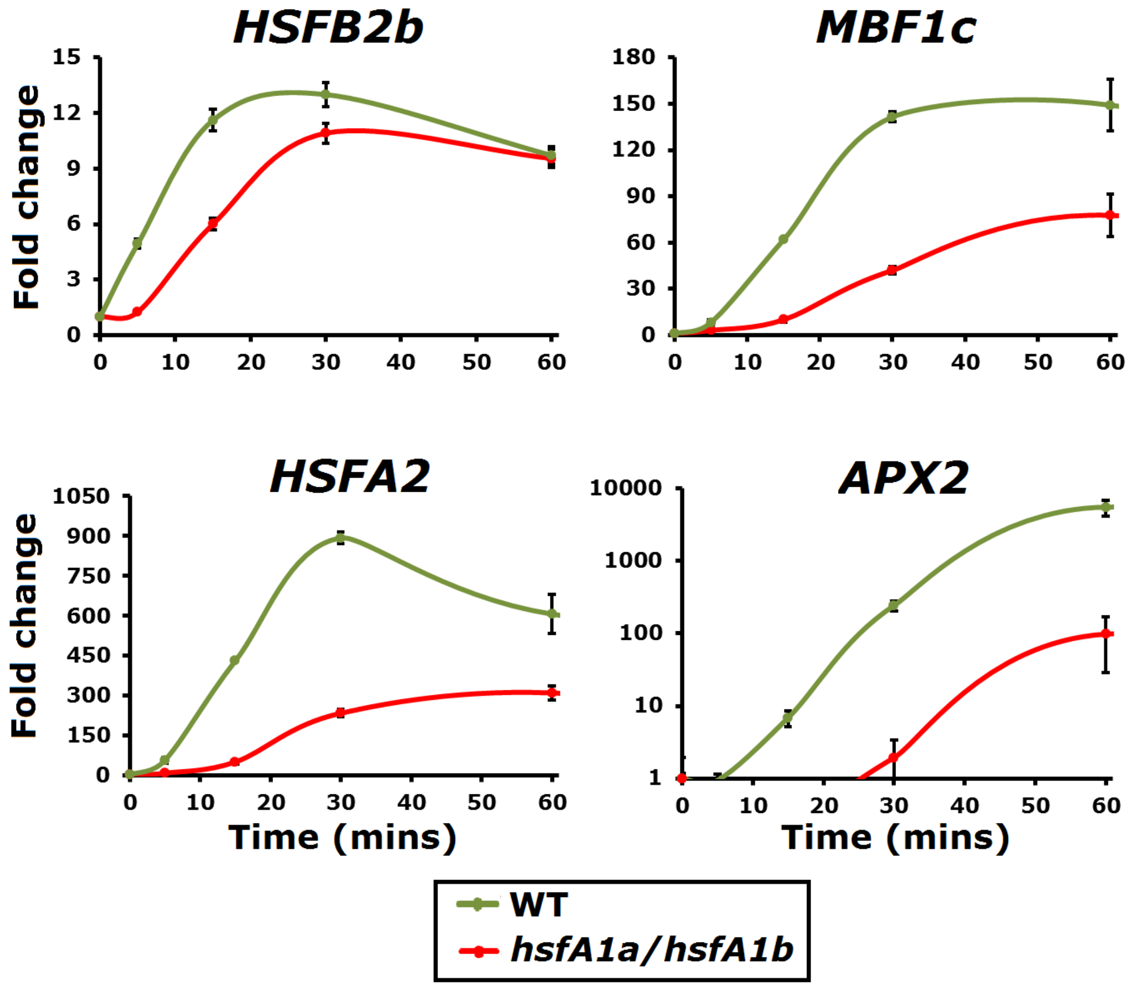

Figure S2. Time-series qRT-PCR results comparing the activation time of four heat-responsive genes, *HSFB2b*, *MBF1c*, *HSFA2*, and *APX2* between wild type (WT; Ws-0) and *hsfA1a/hsfA1b* plants.

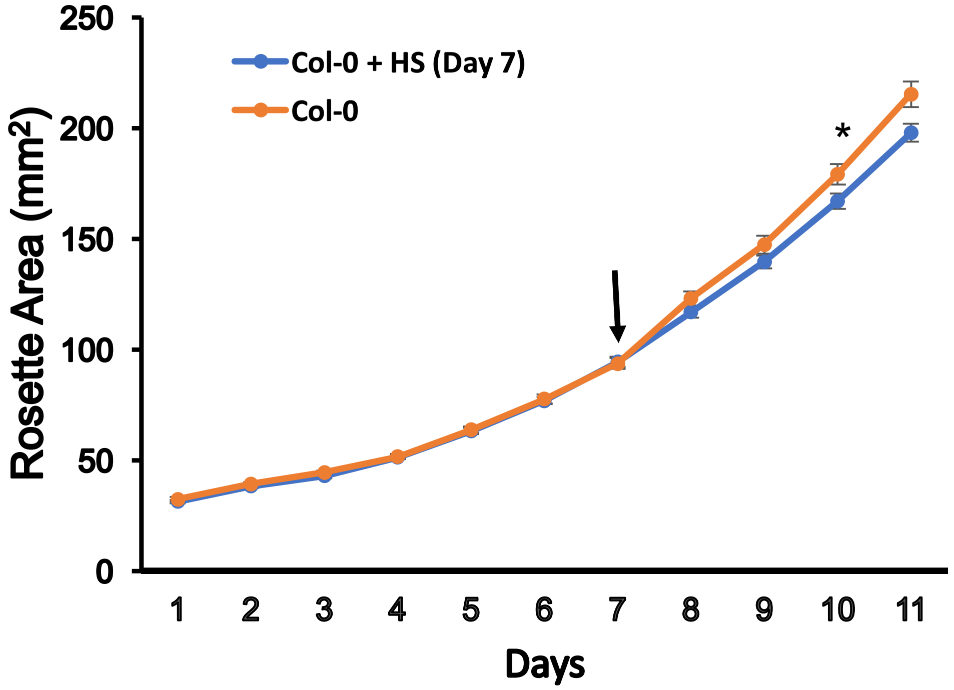

Figure S3. Impact of heat stress on *Arabidopsis* on the increase in rosette area. Rosette area of *Arabidopsis* seedlings were germinated and grown in 8h day: 16h night conditions on soil-filled square plastic pots (3.5cmx3.5cmx5.5cm) for 2 weeks and then their rosette areas measured daily for 11 days using chlorophyll fluorescence imaging (see Methods). **A)** 2 sets of wildtype plants grown without stress (**Col-0**) and heat stress on day 7 (arrowed) for 2 hours at 37°C (**Col-0 + HS (Day 7)**).

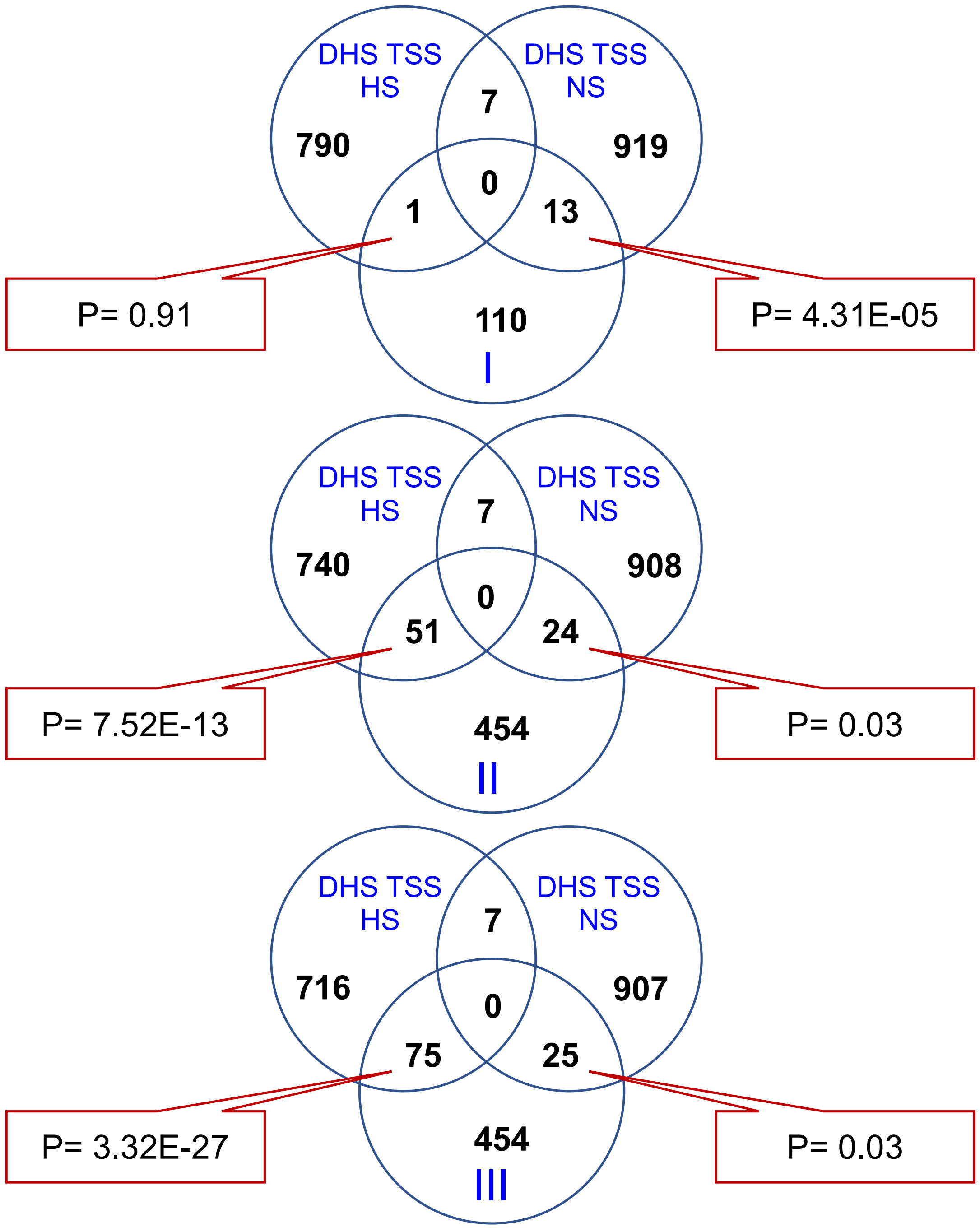

Figure S4. Venn diagrams showing the degree of overlap between Groups I-III HSFA1b target genes and the nearest Transcription Start Site (TSS) loci in genome-mapped DNAse1 hypersensitive sites (DHS) from Sullivan et al., 2014 which used control and HS seedlings. The P values in the callout banners are calculated from a Hypergeometric Distribution Test.

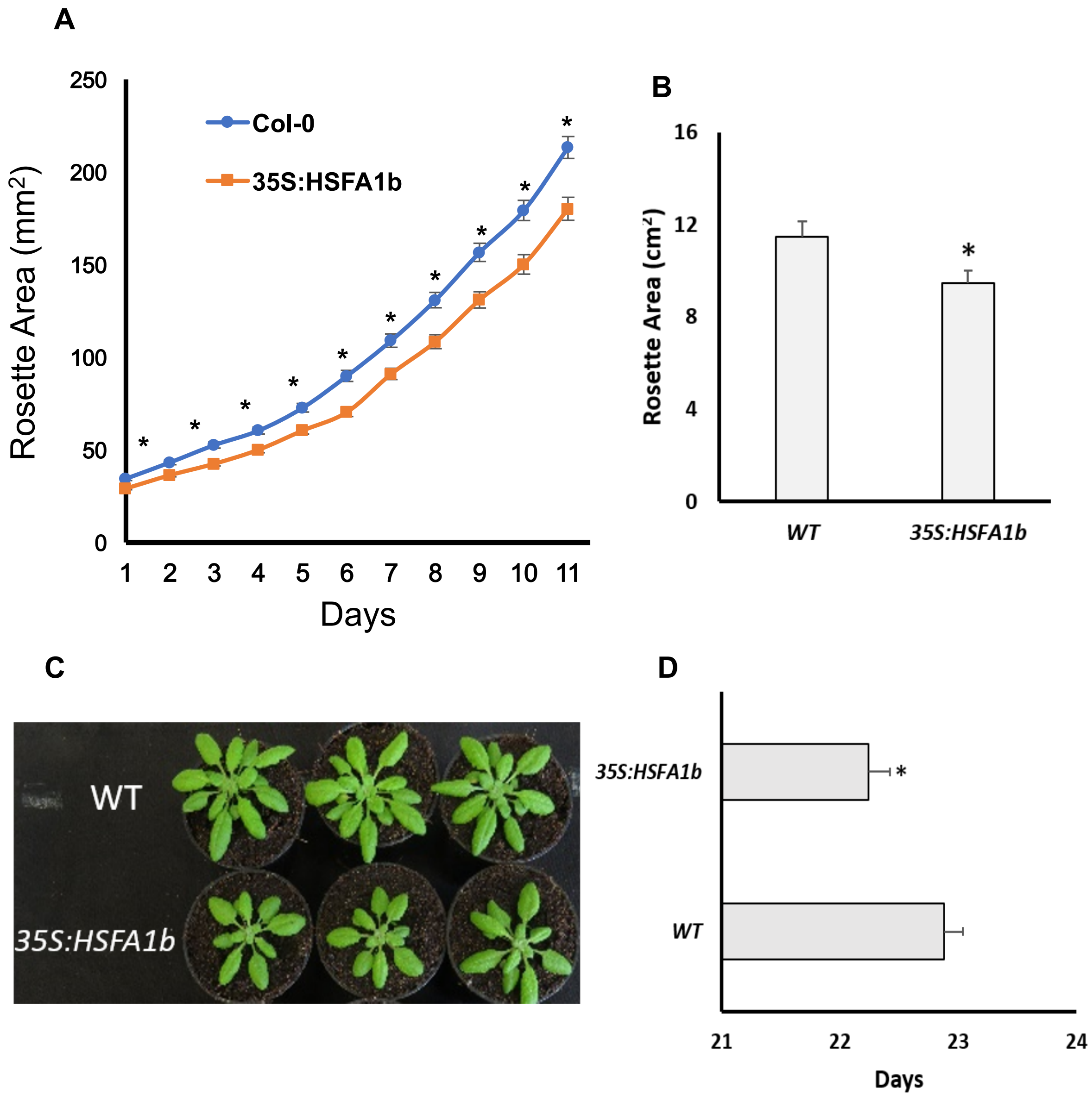

Figure S5. Phenotype of *35S:HSFA1b* plants compared to Col-0. **A)** Rosette area expansion of 15 – 25 days post-germination plants (n=12; single experiment) from days 1-11 of measurements. Areas were determined by imaging the chlorophyll fluoresence parameters F_0 see Methods. **B)** Total rosette area; of 5-week-old plants. **C)** The photograph shows typical examples of rosettes from Col-0 and 35S:HSFA1b plants under stated growth conditions at 4 weeks. **D)** Flowering time i.e. number of days needed to attain a flowering bolt height ≥ 1cm). To induce flowering in a controlled way, 4-week-old plants grown in 8h day: 16h night were transferred to 16h day: 8h night. Asterisks means p < 0.05 (Students t-test; n= 12 plants)

HSFA1b DEGs bound by BZIP28	gene symbols	HSFA1b bound	HSFA1b DEGs bound by BZIP28	gene symbols	HSFA1b bound
AT1G04980	PDIL2-2	yes	AT3G55240	AT3G55240	no
AT1G07670	ECA4	yes	AT3G62610	MYB11	no
AT1G14360	UTR3	yes	AT4G08230	AT4G08230	no
AT1G21750	PDIL1-1	yes	AT4G16660	AT4G16660	yes
AT1G27350	AT1G27350	yes	AT4G21810	DER2.1	no
AT1G72280	ERO1	yes	AT4G24920	AT4G24920	no
AT1G77510	PDIL1-2	no	AT4G29330	DER1	yes
AT2G02810	UTR1	yes	AT4G34620	SSR16	no
AT2G32920	PDIL2-3	yes	AT4G35780	STY17	no
AT2G34420	LHB1B2	yes	AT5G13100	AT5G13100	no
AT2G34430	LHB1B1	no	AT5G17760	AT5G17760	no
AT2G34620	AT2G34620	no	AT5G28540	BIP1	yes
AT3G02470	SAMDC	no	AT5G45630	AT5G45630	yes
AT3G08970	ATERDJ3A	no	AT5G64510	TIN1	yes
AT3G54960	PDIL1-3	no			

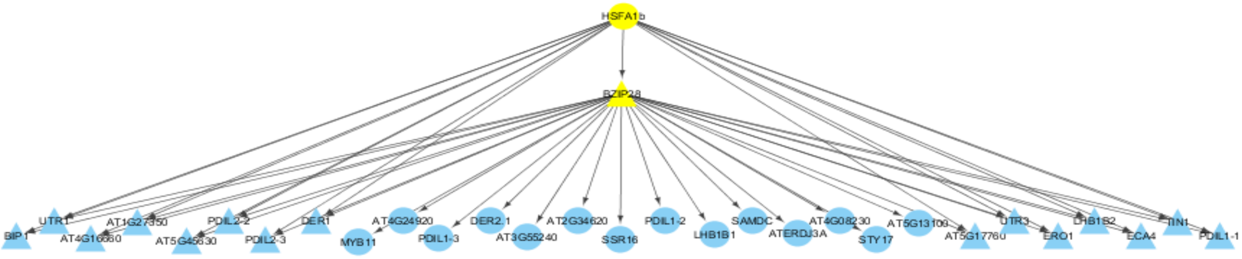

Figure S6 Hierarchical network showing interaction between HSFA1b and BZIP28 from ChIP-seq data (Data S1; Zhang et al 2017) and co-regulated genes for these interactions determined from overlapping RNA-seq data sets (Data S3; Zhang et al 2017). The interactions are summarised in the accompanying table. The network was generated in Cytoscape from a compatible network file generated in Excel.

Table S1 Experimentally confirmed developmental genes bound by HSFA1b.

Those words in black letters are differentially expressed in WT plants subjected to HS. Those in bold are differentially expressed in 35S:HSFA1b plants under NS compared with WT plants under NS and/or HS. Those in blue are bound by HSFA1b but not differentially expressed significantly under HS or in 35S:HSFA1b plants.

Locus Identifier	***Primary Gene Symbol***	***Reference***
**AT1G02090**	**FUSCA 5 (FUS5)**	Karniol B, Malec P, Chamovitz DA. Plant Cell. 1999;11: 839. doi:10.1105/tpc.11.5.839
**AT1G09080**	**BINDING PROTEIN 3 (BIP3)**	Maruyama D, Endo T, Nishikawa S. Plant Signal Behav. 2015;2324: 2. doi:10.1080/15592324.2015.1035853
AT1G13245	ROTUNDIFOLIA LIKE 17 (RTFL17)	Valdivia ER, Chevalier D, Sampedro J, Taylor I, Niederhuth CE, Walker JC. J Exp Bot. 2012;63: 1405. doi:10.1093/jxb/err378
**AT1G14360**	**UDP-GALACTOSE TRANSPORTER 3 (UTR3)**	Reyes F, León G, Donoso M, Brandizzí F, Weber APM, Orellana A. Plant J. 2010;61: 423. doi:10.1111/j.1365-313X.2009.04066.x
**AT1G14740**	**TITANIA 1 (TTA1)**	Lin TF, Saiga S, Abe M, Laux T. PLoS One. 2016;11: 1. doi:10.1371/journal.pone.0155657
**AT1G18330**	**REVIELLE 7 (RVE7)**	Kuno N, Moller S, Shinomura T, Xu X, Chua N-H, Furuya M. Plant Cell. 2003;15: 2476. doi:10.1105/tpc.014217
**AT1G19180**	**JASMONATE-ZIM-DOMAIN PROTEIN 1 (JAZ1)**	Song S, Qi T, Fan M, Zhang X, Gao H, Huang H, et al. PLoS Genet. 2013;9: e1003653. doi:10.1371/journal.pgen.1003653
**AT1G20780**	**SENESCENCE-ASSOCIATED E3 UBIQUITIN LIGASE 1 (SAUL1)**	Raab S, Drechsel G, Zarepour M, Hartung W, Koshiba T, Bittner F, et al. Plant J. 2009;59: 39. doi:10.1111/j.1365-313X.2009.03846.x
**AT1G21750**	**PDI-LIKE 1-1 (PDIL1-1)**	Kumar MN, Hsieh Y, Verslues PE. Proc Natl Acad Sci. 2015;112: 10545. doi:10.1073/pnas.1510140112

**AT1G32640**	**MYC2**	Valenzuela CE, Acevedo-Acevedo O, Miranda GS, Vergara-Barros P, Holuigue L, Figueroa CR, et al. J Exp Bot. 2016;67: 4209. doi:10.1093/jxb/erw202
**AT1G33140**	**PIGGYBACK 2 (PGY2)**	Pinon V, Etchells JP, Rossignol P, Collier S a, Arroyo JM, Martienssen R a, et al. Development. 2008;135: 1315. doi:10.1242/dev.016469
AT1G34245	EPIDERMAL PATTERNING FACTOR 2 (EPF2)	Hunt L, Gray JE. Curr Biol. Elsevier Ltd; 2009;19: 864–869. doi:10.1016/j.cub.2009.03.069
AT1G48410	ARGONAUTE 1 (AGO1)	Bohmert K, Camus I, Bellini C, Bouchez D, Caboche M, Banning C. EMBO J. 1998;17: 170. doi:10.1093/emboj/17.1.170
**AT1G60190**	**PLANT U-BOX 19 (PUB19)**	Moon J, Parry G, Estelle M. Plant Cell. 2004;16: 3181. doi:10.1105/tpc.104.161220.3182
**AT1G66400**	**CALMODULIN LIKE 23 (CML23)**	Tsai, Y-C, Delk, NA, Chowdhury, NI, Braam J. Plant Signal Behav. 2007;2: 446. doi:10.4161/psb.2.6.4695
**AT1G67960**	**POLLEN DEFECTIVE IN GUIDANCE 1 (POD1)**	Li H-J, Xue Y, Jia D-J, Wang T, Hi D-Q, Liu J, et al. Plant Cell. 2011;23: 3288. doi:10.1105/tpc.111.088914
AT1G69530	EXPANSIN A1 (EXPA1)	Esmon CA, Tinsley AG, Ljung K, Sandberg G, Hearne LB, Liscum E A. Proc Natl Acad Sci. 2005;103: 236. doi:10.1073/pnas.050127103
**AT1G70060**	**SIN3-LIKE 4 (SNL4)**	Bowen AJ, Gonzalez D, Mullins JGL, Bhatt AM, Martinez A, Conlan RS. J Mol Biol. 2010;395: 937. doi:10.1016/j.jmb.2009.11.065
**AT1G70140**	**FORMIN 8 (FH8)**	Xue X, Guo C, Du F, Lu Q, Zhang C, Ren H. Mol Plant 2011;4: 264. doi:10.1093/mp/ssq085
AT1G78080	RELATED TO AP2 4 (RAP2.4)	Iwase A, Harashima H, Ikeuchi M, Rymen B, Ohnuma M, Komaki S, et al. Plant Cell. 2016;29: tpc.00623.2016. doi:10.1105/tpc.16.00623
AT1G80490	TOPLESS-RELATED 1 (TPR1)	Oh E, Zhu J-Y, Ryu H, Hwang I, Wang Z-Y. Nat Commun. 2014;5: 4140. doi:10.1038/ncomms5140

**AT2G02760**	**UBIQUITING-CONJUGATING ENZYME 2 (UBC2)**	Xu L, Ménard R, Berr A, Fuchs J, Cognat V, Meyer D, et al. Plant J. 2009;57: 279. doi:10.1111/j.1365-313X.2008.03684.x
**AT2G02810**	**UDP-GALACTOSE TRANSPORTER 1 (UTR1)**	Reyes F, León G, Donoso M, Brandizzí F, Weber APM, Orellana A. Plant J. 2010;61: 423. doi:10.1111/j.1365-313X.2009.04066.x
**AT2G03120**	**SIGNAL PEPTIDE PEPTIDASE (SPP)**	Han S, Green L, Schnell DJ. Plant Physiol. 2009;149: 1289. doi:10.1104/pp.108.130252
**AT2G04030**	**(CR88)**	Feng J, Fan P, Jiang P, Lu S, Chen X, Li Y. Physiol Plant. 2014;150: 292. doi:10.1111/ppl.12083
**AT2G14120**	**DYNAMIN RELATED PROTEIN (DRP3B)**	Aung K, Hu J. J Integr Plant Biol. 2012;54: 921. doi:10.1111/j.1744-7909.2012.01174.x
**AT2G15790**	**SQUINT (SQN)**	Berardini TZ, Bollman K, Sun H, Poethig RS. Science (80-). 2001;291: 2405. doi:10.1126/science.1057144
**AT2G19580**	**TETRASPANIN 2 (TET2)**	Cnops G, Neyt P, Raes J, Petrarulo M, Nelissen H, Malenica N, et al. Plant Cell. 2006;18: 852. doi:10.1105/tpc.105.040568
**AT2G21660**	**GLYCINE-RICH RNA-BINDING PROTEIN 7 (GRP7)**	Xiao J, Li C, Xu S, Xing L, Xu Y, Chong K. Plant Physiol. 2015;169: 2102. doi:10.1104/pp.15.00801
**AT2G23430**	**(ICK1)**	Malinowski R, Kasprzewska A, Fleming AJ. Plant J. 2011;66: 941. doi:10.1111/j.1365-313X.2011.04559.x
**AT2G28000**	**CHAPERONIN-60ALPHA (CPN60A)**	Apuya NR, Yadegari R, Fischer RL, Harada JJ, Zimmerman JL, Goldberg RB. Plant Physiol. 2001;126: 717. doi:10.1104/pp.126.2.717
**AT2G36740**	**(SWC2)**	Choi K, Park C, Lee J, Oh M, Noh B, Lee I. Development. 2007;134: 1931. doi:10.1242/dev.001891
**AT2G41940**	**ZINC FINGER PROTEIN 8 (ZFP8)**	Zhou Z, An L, Sun L, Gan Y. Plant Signal Behav. 2012;7: 28. doi:10.4161/psb.7.1.18404134.
**AT2G42590**	**GENERAL REGULATORY FACTOR 9 (GRF9)**	Mayfield JD, Paul AL, Ferl RJ.. J Exp Bot. 2012;63: 3061. doi:10.1093/jxb/ers022

**AT2G42620**	**MORE AXILLARY BRANCHES 2 (MAX2)**	Wang L, Wang B, Jiang L, Liu X, Li X, Lu Z, et al. Plant Cell. 2015;27: 1. doi:10.1105/tpc.15.00605
**AT3G08590**	**2,3-BIPHOSPHOGLYCERATE-INDEPENDENT PHOSPHOGLYCERATE MUTASE 2 (iPGAM2)**	Zhao Z, Assmann SM. J Exp Bot. 2011;62: 5179. doi:10.1093/jxb/err223137.
**AT3G09840**	**CELL DIVISION CYCLE 48 (CDC48)**	Copeland C, Woloshen V, Huang Y, Li X. Plant J. 2016;88: 294. doi:10.1111/tpj.13251
**AT3G29030**	**EXPANSIN A5 (EXPA5)**	Bergonci T, Silva-Filho MC, Moura DS. Plant Signal Behav. 2014;9: e976146. doi:10.4161/15592324.2014.976146
**AT3G44110**	**(J3)**	Shen L, Yu H. Plant Signal Behav. 2011;6: 601. doi:10.4161/psb.6.4.15375
**AT3G50060**	**MYB DOMAIN PROTEIN 77 (MYB77)**	Zhao Y, Xing L, Wang X, Hou Y-J, Gao J, Wang P, et al. Sci Signal. 2014;7: ra53-ra53. doi:10.1126/scisignal.2005051
AT3G51840	ACYL-COA OXIDASE 4 (ACX4)	Rylott EL, Rogers CA, Gilday AD, Edgell T, Larson TR, Graham IA. J Biol Chem. 2003;278: 21370. doi:10.1074/jbc.M300826200
**AT3G54920**	**POWDERY MILDEW RESISTANT 6 (PMR6)**	Vogel JP, Raab TK, Schiff C, Somerville SC. Plant Cell. 2002;14: 2095. doi:10.1105/tpc.003509
**AT3G59060**	**PHYTOCHROME INTERACTING FACTOR 3-LIKE 6 (PIL6)**	Pedmale U V., Huang SSC, Zander M, Cole BJ, Hetzel J, Ljung K, et al. Cell 2016;164: 233. doi:10.1016/j.cell.2015.12.018
**AT3G63440**	**CYTOKININ OXIDASE/DEHYDROGENASE 6 (CKX6)**	Lall S, Nettleton D, DeCook R, Che P, Howell SH. Genetics. 2004;167:1892. doi:10.1534/genetics.103.025213
**AT4G00060**	**MATERNAL EFFECT EMBRYO ARREST 44 (MEE44)**	Pagnussat GC, Yu H-J, Ngo Q a, Rajani S, Mayalagu S, Johnson CS, et al. Development. 2005;132: 603. doi:10.1242/dev.01595
AT4G01250	WRKY22	Zhou X, Jiang Y, Yu D. 2011;31: 303. doi:10.1007/s10059-011-0047-1
**AT4G02980**	**ENDOPLASMIC RETICULUM AUXIN BINDING PROTEIN 1 (ABP1)**	Grones P, Chen X, Simon S, Kaufmann WA, De Rycke R, Nodzyski T, et al. J Exp Bot. 2015;66: 5055. doi:10.1093/jxb/erv177

**AT4G05420**	**DAMAGED DNA BINDING PROTEIN 1A (DDB1A)**	Bjerkan KN, Grini PE. Plant Signal Behav. 2013;2324: 1. doi:10.4161/psb.25347
**AT4G08920**	**CRYPTOCHROME 1 (CRY1)**	Pedmale U V., Huang SSC, Zander M, Cole BJ, Hetzel J, Ljung K, et al. Cell 2016;164: 233. doi:10.1016/j.cell.2015.12.018
**AT4G09510**	**CYTOSOLIC INVERTASE 2 (CINV2)**	Barratt DHP, Derbyshire P, Findlay K, Pike M, Wellner N, Lunn J, et al. Proc Natl Acad Sci U S A. 2009;106: 13124. doi:10.1073/pnas.0900689106
**AT4G11660**	**HSFB2b**	Kolmos E, Chow BY, Pruneda-Paz JL, Kay SA. Proc Natl Acad Sci. 2014;111: 16172. doi:10.1073/pnas.1418483111
**AT4G20260**	**PLASMA-MEMBRANE ASSOCIATED CATION-BINDING PROTEIN 1 (PCAP1)**	Li J, Wang X, Qin T, Zhang Y, Liu X, Sun J, et al. Plant Cell. 2011;23: 4427. doi:10.1105/tpc.111.092684
**AT4G22950**	**AGAMOUS-LIKE 19 (AGL19)**	Kang MJ, Jin HS, Noh YS, Noh B. New Phytol. 2015;206: 281. doi:10.1111/nph.13161
**AT4G24190**	**SHEPHERD (SHD)**	Klein EM, Mascheroni L, Pompa A, Ragni L, Weimar T, Lilley KS, et al. Plant J. 2006;48: 673. doi:10.1111/j.1365-313X.2006.02904.x
**AT4G24280**	**CHLOROPLAST HEAT SHOCK PROTEIN 70-1 (cpHsc70-1)**	Su P-H, Li H-M. Plant Physiol. 2008;146: 1241. doi:10.1104/pp.107.114496
AT4G25520	SEUSS-LIKE 1 (SLK1)	Bao F, Azhakanandam S, Franks RG. Plant Physiol. 2010;152: 836. doi:10.1104/pp.109.146183
AT4G32410	CELLULOSE SYNTHASE 1 (CESA1)	Chen S, Ehrhardt DW, Somerville CR. Proc Natl Acad Sci. 2010;107: 17193. doi:10.1073/pnas.1012348107
AT4G34460	GTP BINDING PROTEIN BETA 1 (AGB1)	Ishida T, Tabata R, Yamada M, Aida M, Mitsumasu K, Fujiwara M, et al. EMBO Rep. 2014;15: 1202. doi:10.15252/embr.201438660
**AT5G02500**	**HEAT SHOCK COGNATE PROTEIN 70-1 (HSC70-1)**	Cazalé AC, Clément M, Chiarenza S, Roncato MA, Pochon N, Creff A, et al. J Exp Bot. 2009;60: 2653–2664. doi:10.1093/jxb/erp109

AT5G04410	NAC DOMAIN CONTAINING PROTEIN 2 (NAC2)	Nguyen HM, Schippers JHM, Gõni-Ramos O, Christoph MP, Dortay H, Van Der Hoorn RAL, et al. Plant J. 2013;74: 25. doi:10.1111/tpj.12097
AT5G05690	CONSTITUTIVE PHOTOMORPHOGENIC DWARF (CPD)	Zhiponova MK, Vanhoutte I, Boudolf V, Betti C, Dhondt S, Coppens F, et al. New Phytol. 2013;197: 490. doi:10.1111/nph.12036
AT5G08790	ATAF2	Peng H, Zhao J, Neff MM. Development. 2015; 4129. doi:10.1242/dev.124347
**AT5G09810**	**ACTIN 7 (ACT7)**	Mei Y, Gao H-B, Yuan M, Xue H-W. Plant Cell. 2012;24: 1066. doi:10.1105/tpc.111.095059
**AT5G15470**	**GALACTURONOSYLTRANSFERASE 14 (GAUT14)**	Wang L, Wang W, Wang Y-Q, Liu Y, Wang J, Zhang X, et al. Mol Plant. 2013;6: 1131. doi:10.1093/mp/sst084
**AT5G17300**	**REVEILLE 1 (RVE1)**	Xu G, Guo H, Zhang D, Chen D, Jiang Z, Lin R. Photosynth Res. 2015;126: 331–340. doi:10.1007/s11120-015-0146-5
**AT5G17490**	**RGA-LIKE PROTEIN 3 (RGL3)**	Achard P, Renou J-P, Berthomé R, Harberd NP, Genschik P. Curr Biol. 2008;18: 656. doi:10.1016/j.cub.2008.04.034
AT5G23670	LONG CHAIN BASE2 (LCB2)	Teng C, Dong H, Shi L, Deng Y, Mu J, Zhang J, et al. Plant Physiol. 2008;146: 1322. doi:10.1104/pp.107.113506
**AT5G28540**	**BIP1**	Maruyama D, Endo T, Nishikawa S. Proc Natl Acad Sci. 2010;107: 1689. doi:10.1073/pnas.0905795107
**AT5G42020**	**BIP2**	Maruyama D, Endo T, Nishikawa S. Proc Natl Acad Sci. 2010;107: 1689. doi:10.1073/pnas.0905795107
**AT5G46700**	**TORNADO 2 (TRN2)**	Cnops G, Neyt P, Raes J, Petrarulo M, Nelissen H, Malenica N, et al. Plant Cell. 2006;18: 852. doi:10.1105/tpc.105.040568
**AT5G48030**	**GAMETOPHYTIC FACTOR 2 (GFA2)**	Christensen C, Subramanian S, Drews GN. Dev Biol. 1998;202: 136. doi:10.1006/dbio.1998.8980
**AT5G53400**	**BOBBER1 (BOB1)**	Kaplinsky NJ. Plant Signal Behav. 2009;4: 1157. doi:10.1104/pp.109.142125

**AT5G54510**	**DWARF IN LIGHT 1 (DFL1)**	Nakazawa M, Yabe N, Ichikawa T, Yamamoto YY, Yoshizumi T, Hasunuma K, et al. Plant J. 2001;25: 213. doi:10.1046/j.1365-313X.2001.00957.x
AT5G55920	OLIGOCELLULA 2 (OLI2)	Fujikura U, Horiguchi G, Ponce MR, Micol JL, Tsukaya H. Plant J. 2009;59: 499. doi:10.1111/j.1365-313X.2009.03886.x
**AT5G56600**	**PROFILIN 3 (PRF3)**	Fan T, Zhai H, Shi W, Wang J, Jia H, Xiang Y, et al. Plant Cell Rep. 2013;32: 149. doi:10.1007/s00299-012-1349-2
**AT5G62020**	**HSFB2a**	Wunderlich M, Groß-Hardt R, Schöffl F. Plant Mol Biol. 2014;85: 541. doi:10.1007/s11103-014-0202-0
**AT5G62090**	**SEUSS-LIKE 2 (SLK2)**	Bao F, Azhakanandam S, Franks RG. Plant Physiol. 2010;152: 836. doi:10.1104/pp.109.146183
AT5G62430	CYCLING DOF FACTOR 1 (CDF1)	Song YH, Smith RW, To BJ, Millar AJ, Imaizumi T. Science (80-). 2012;336: 1045. doi:10.1126/science.1219644
**AT5G64510**	**TUNICAMYCIN INDUCED 1 (TIN1)**	Iwata Y, Nishino T, Iwano M, Takayama S, Koizumi N. Plant Biotechnol. 2012;29: 51. doi:10.5511/plantbiotechnology.11.1228a
AT5G67420	LOB DOMAIN-CONTAINING PROTEIN 37 (LBD37)	Shuai, B. Reynaga-Pena, CG, Springer P. Plant Physiol. 2002;129: 747. doi:10.1104/pp.010926
AT1G04400	CRYPTOCHROME 2 (CRY2)	Pedmale U V., Huang SSC, Zander M, Cole BJ, Hetzel J, Ljung K, et al. Cell 2016;164: 233. doi:10.1016/j.cell.2015.12.018
AT1G10470	RESPONSE REGULATOR 4 (ARR4)	Verma V, Sivaraman J, Srivastava AK, Sadanandom A, Kumar PP. New Phytol. 2015;206: 726. doi:10.1111/nph.13297
AT1G11130	STRUBBELIG (SUB)	Kwak S-HH, Woo S, Lee MM, Schiefelbein J. Plant Physiol. 2014;166: 987. doi:10.1104/pp.114.247288
AT1G14000	VH1-INTERACTING KINASE (VIK)	Ceserani T, Trofka A, Gandotra N, Nelson T. Plant J. 2009;57: 1000. doi:10.1111/j.1365-313X.2008.03742.x

AT1G18040	CYCLIN-DEPENDENT KINASE D1;3 (CDKD1;3)	Takatsuka H, Umeda-Hara C, Umeda M. Plant J. 2015;82: 1004. doi:10.1111/tpj.12872
AT1G35580	CYTOSOLIC INVERTASE 1 (CINV1)	Barratt DHP, Derbyshire P, Findlay K, Pike M, Wellner N, Lunn J, et al. Proc Natl Acad Sci U S A. 2009;106: 13124. doi:10.1073/pnas.0900689106
AT1G66740	SGA2	Lario L, Ramirez-Parra E, Gutierrez C, Spampinato C, Casati P. Plant Physiol. 2013;162: 1164. doi:10.1104/pp.112.212837
AT1G71040	LOW PHOSPHATE ROOT2 (LPR2)	Ticconi CA, Lucero RD, Sakhonwasee S, Adamson AW, Creff A, Nussaume L, et al. Proc Natl Acad Sci. 2009;106: 14174. doi:10.1073/pnas.0901778106
AT1G72440	EMBRYO SAC DEVELOPMENT ARREST 25 (EDA25)	Li N, Yuan L, Liu N, Shi D, Li X, Tang Z, et al. Plant Physiol. 2009;151: 1486. doi:10.1104/pp.109.142414
AT1G80080	TOO MANY MOUTHS (TMM)	Geisler M, Yang M, Sack FD. Planta. 1998;205: 522. doi:10.1007/s004250050351
**AT2G01420**	**PIN-FORMED 4 (PIN4)**	Friml J, Benková E, Blilou I, Wisniewska J, Hamann T, Ljung K, et al. Cell. 2002;108: 661. doi:10.1016/S0092-8674(02)00656-6
AT2G14210	AGAMOUS-LIKE 44 (AGL44)	Gan Y, Bernreiter A, Filleur S, Abram B, Forde BG. Plant Cell Physiol. 2012;53: 1003. doi:10.1093/pcp/pcs050
AT2G24840	AGAMOUS-LIKE 61 (AGL61)	Steffen JG, Kang I-H, Portereiko MF, Lloyd A, Drews GN. Plant Physiol. 2008;148: 258. doi:10.1104/pp.108.119404
**AT2G28056**	**MICRORNA172A (MIR172A)**	Huo H, Wei S, Bradford KJ. Proc Natl Acad Sci. 2016;1: 1. doi:10.1073/pnas.1600558113
AT2G28550	RELATED TO AP2.7 (RAP2.7)	Zhang B, Wang L, Zeng L, Zhang C, Ma H. Genes Dev. 2015;29: 975. doi:10.1101/gad.251520.114.
AT2G38120	AUXIN RESISTANT 1 (AUX1)	Kakani A, Li G, Peng Z. Planta. 2009;229: 645. doi:10.1007/s00425-008-0846-6
AT2G39705	ROTUNDIFOLIA LIKE 8 (RTFL8)	Wen J, Lease KA, Walker JC. Plant J. 2004;37: 668. doi:10.1111/j.1365-313X.2003.01994.x

AT2G39990	EUKARYOTIC TRANSLATION INITIATION FACTOR 2 (EIF2)	Xia C, Wang Y, Li W, Chen Y-R, Deng Y, Zhang X, et al. Plant J. 2010;63: 189. doi:10.1111/j.1365-313X.2010.04237.x
AT2G44810	DEFECTIVE ANTHER DEHISCENCE 1 (DAD1)	Kim SG, Lee S, Kim YS, Yun DJ, Woo JC, Park CM. Plant Mol Biol. 2010;74: 337. doi:10.1007/s11103-010-9677-5
AT2G45660	AGAMOUS-LIKE 20 (AGL20)	Richter R, Bastakis E, Schwechheimer C. Plant Physiol. 2013;162: 1992. doi:10.1104/pp.113.219238
AT2G46410	CAPRICE (CPC)	Tominaga-Wada R, Wada T. J Plant Physiol. 2016;199: 111. doi:10.1016/j.jplph.2016.05.014
AT3G07780	OBERON1 (OBE1)	Saiga S, Furumizu C, Yokoyama R, Kurata T, Sato S, Kato T, et al. Development. 2008;135: 1751. doi:10.1242/dev.014993
**AT3G22380**	**TIME FOR COFFEE (TIC)**	Hong LW, Yan DW, Liu WC, Chen HG, Lu YT. J Exp Bot. 2014;65: 275. doi:10.1093/jxb/ert374
AT3G48360	BTB AND TAZ DOMAIN PROTEIN 2 (bt2)	Robert HS, Quint A, Brand D, Vivian-Smith A, Offringa R. Plant J. 2009;58: 109. doi:10.1111/j.1365-313X.2008.03764.x
AT3G54340	APETALA 3 (AP3)	Jack T, Fox GL, Meyerowitz EM. Cell. 1994;76: 703. doi:10.1016/0092-8674(94)90509-6
**AT3G63190**	**RIBOSOME RECYCLING FACTOR, CHLOROPLAST PRECURSOR (RRF)**	Wang L, Ouyang M, Li Q, Zou M, Guo J, Ma J, et al. Plant Mol Biol. 2010;74: 47. doi:10.1007/s11103-010-9653-0
AT4G01370	MAP KINASE 4 (MPK4)	Gawronski P, Witon D, Vashutina K, Bederska M, Betlinski B, Rusaczonek A, et al. Mol Plant. 2014;7: 1151. doi:10.1093/mp/ssu060
**AT4G15560**	**CLOROPLASTOS ALTERADOS 1 (CLA1)**	Mandel MA, Feldmann K a, Herrera-Estrella L, Rocha-Sosa M, Leon P. Plant J. 1996;9: 649. doi:10.1046/j.1365-313X.1996.9050649.x
AT4G25350	SHORT HYPOCOTYL UNDER BLUE1 (SHB1)	Zhou Y, Ni M. Dev Biol. 2009;331: 50. doi:10.1016/j.ydbio.2009.04.023198.
AT4G25640	DETOXIFYING EFFLUX CARRIER 35 (DTX35)	Thompson EP, Wilkins C, Demidchik V, Davies JM, Glover BJ. J Exp Bot. 2010;61: 439. doi:10.1093/jxb/erp312

AT4G26690	SHAVEN 3 (SHV3)	Yeats TH, Sorek H, Wemmer DE, Somerville CR. Plant Physiol. 2016;171: 110. doi:10.1104/pp.16.00302
AT4G30960	SOS3-INTERACTING PROTEIN 3 (SIP3)	Tripathi V, Parasuraman B, Laxmi A, Chattopadhyay D. Plant J. 2009;58: 778. doi:10.1111/j.1365-313X.2009.03812.x
AT4G34160	CYCLIN D3;1 (CYCD3;1)	Randall RS, Sornay E, Dewitte W, Murray JAH. J Exp Bot. 2015;66: 3991. doi:10.1093/jxb/erv200
AT4G38430	RHO GUANYL-NUCLEOTIDE EXCHANGE FACTOR 1 (ROPGEF1)	Liu Y, Dong, Q, Kita, D, Huang, J-B, Liu, G, Wu, X, Zhu, X, Cheung, AY, Wu, H-M, Tao L-Z. Plant Physiol. 2017; doi:10.1104/pp.17.00697
AT4G39800	D-MYO-INOSITOL 3-PHOSPHATE SYNTHASE 1 (MIPS1)	Chen H, Xiong L. J Biol Chem. 2010;285: 24238. doi:10.1074/jbc.M110.123661
**AT5G01240**	**LIKE AUXIN RESISTANT 1 (LAX1)**	Kasprzewska A, Carter R, Swarup R, Bennett M, Monk N, Hobbs JK, et al. Plant J. 2015;83: 705. doi:10.1111/tpj.12921
AT5G01270	CARBOXYL-TERMINAL DOMAIN (CTD) PHOSPHATASE-LIKE 2 (CPL2)	Ueda A, Li P, Feng Y, Vikram M, Kim S, Kang CH, et al. Plant Mol Biol. 2008;67: 683. doi:10.1007/s11103-008-9348-y
AT5G02030	REPLUMLESS (RPL)	Bencivenga S, Serrano-Mislata A, Bush M, Fox S, Sablowski R. Dev Cell. 2016;39: 198. doi:10.1016/j.devcel.2016.08.013
AT5G07290	MEI2-LIKE 4 (ML4)	Kaur J, Sebastian J, Siddiqi I. Plant Cell. 2006;18: 545. doi:10.1105/tpc.105.039156.1
AT5G09970	CYTOCHROME P450, FAMILY 78, SUBFAMILY A, POLYPEPTIDE 7 (CYP78A7)	Eriksson S, Stransfeld L, Adamski NM, Breuninger H, Lenhard M. Curr Biol. 2010;20: 527. doi:10.1016/j.cub.2010.01.039
AT5G24860	FLOWERING PROMOTING FACTOR 1 (FPF1)	Kania T, Russenberger D, Peng S, Apel K, Melzer S. Plant Cell. 1997;9: 1327. doi:10.1105/tpc.9.8.1327
AT5G41990	WITH NO LYSINE (K) KINASE 8 (WNK8)	Wang, Y, Liu, K, Liao, H, Zhuang, C., Yan X. Plant Biol. 2008; 8. doi:10.1111/j.1438-8677.2008.00072.x
AT5G51760	ABA-HYPERSENSITIVE GERMINATION 1 (AHG1)	Née G, Kramer K, Nakabayashi K, Yuan B, Xiang Y, Miatton E, et al. Nat Commun. 2017;8: 72. doi:10.1038/s41467-017-00113-6

AT5G61960	MEI2-LIKE PROTEIN 1 (ML1)	Kaur J, Sebastian J, Siddiqi I. Plant Cell. 2006;18: 545. doi:10.1105/tpc.105.039156.1
AT5G62000	AUXIN RESPONSE FACTOR 2 (ARF2)	Schruff M, Spielman M, Tiwari S, Adams S, Fenby N, Scott R. Development. 2006;133: 251. doi:10.1242/dev.02194

Table S2 Primers used in the qPCR analyses.

Primer Name	Sequence (5' to 3')
CDF1_F	CAACGTAAACCAACCTCGCC
CDF1_R	CACTTCTCATGGTCCCACCT
MYB16_F	AGGAAACAGATGGTCAGCGA
MYB16_R	CACTAACCGTTTCTTCAAATGAGTG
LZF1_F	AGGAGATTTTCGGGCTAACCG
LZF1_R	GTTTCATCTTGAGAACGTCTGTCT
NAT_LZF1_F	GGATTAGAGAGGCCATAAACCAG
NAT_LZF1_R	CCAGATGCTTCCTGTACACAC
NAT_MYB16_F	CATTGCCTGAGAAAGCTGGT
NAT_MYB16_R	CATCGATGGAGACCTGAGAAGAG
NAT_CDF1_F	CGCTCACCTTTATTGGTTTCAGT
NAT_CDF1_R	GTTGGTGAACCAGAGGTTGC
HSFB2a_F	CGATGGGAGTTTTCAAACGA
HSFB2a_R	ACAACCATCGTCTGGTTTCG
HSFB2b_F	GGGGTTTCTATTGGGGTCAA
HSFB2b_R	CCATTGGCTCTGCCTTAACA
SZF1_F	TGTTGCTGGCTGTTCTGTGA
SZF1_R	GCTTTCCTCCTCGGACTAGC
GBF3_F	ATGACGTGGTCATCGTCTTG
GBF3_R	CCAGAGCGAAAAAGAGTTCAG
bZIP28_F	ACGACCAAGTTCGTTGAGCA
bZIP28_R	AAACCCCTTGCTTTCTCGCT
RVE1_F	ATGCACCCAAGGTACGGAAG
RVE1_R	TATTCGTCTCCAAGCTCGCC
RVE7_F	CGCGGAAGAATCTCACAAACCAT
RVE7_R	GCATCCCTGAGTAGTGATTCTCC
